# Supplementary material for: Quality of prescribing in older people from a broad family physician perspective: a descriptive pilot study
Source: BMJ Open. 2019 Jun 2;9(6):e027290. doi: 10.1136/bmjopen-2018-027290 (PMC6549657; doi:10.1136/bmjopen-2018-027290)
Supplement: Author's manuscript [file bmjopen-2018-027290.draft_revisions.pdf]

BMJ Open is committed to open peer review. As part of this commitment we make the peer review history of every article we publish publicly available.

When an article is published we post the peer reviewers' comments and the authors' responses online. We also post the versions of the paper that were used during peer review. These are the versions that the peer review comments apply to.

The versions of the paper that follow are the versions that were submitted during the peer review process. They are not the versions of record or the final published versions. They should not be cited or distributed as the published version of this manuscript.

BMJ Open is an open access journal and the full, final, typeset and author-corrected version of record of the manuscript is available on our site with no access controls, subscription charges or pay-per-view fees (http://bmjopen.bmj.com).

If you have any questions on BMJ Open's open peer review process please email info.bmjopen@bmj.com

BMJ Open

Prescribing practices in primary care from a physician perspective: A descriptive pilot study

Journal:	*BMJ Open*
Manuscript ID	bmjopen-2018-027290
Article Type:	Research
Date Submitted by the Author:	15-Oct-2018
Complete List of Authors:	Parodi Lopez, Naldy; Närhälsan Vårgårda Health Centre; Sahlgrenska Academy, University of Gothenburg, Department of Pharmacology<br>Wallerstedt, Susanna; Sahlgrenska Academy, University of Gothenburg, Department of Pharmacology; Sahlgrenska universitetssjukhuset, HTA-centrum
Keywords:	Aged, Inappropriate prescribing, Medication review, Pharmacoepidemiology, Prescription drugs, Primary health care

SCHOLARONE™
Manuscripts

Prescribing practices in primary care from a physician perspective: A descriptive pilot study

Naldy Parodi Lopez[1,2], Susanna M. Wallerstedt[2,3]

[1]Närhälsan Vårgårda Health Centre, Sweden

[2]Department of Pharmacology, Sahlgrenska Academy, University of Gothenburg, Gothenburg, Sweden

[3]HTA-centrum, Sahlgrenska University Hospital, Gothenburg, Sweden

Corresponding author:

Naldy Parodi Lopez, Närhälsan Vårgårda Health Centre, Centrumgatan 2, 44731, Vårgårda, Sweden. Tel.:+460104358532; E-mail: naldy.parodi.lopez@vgregion.se

Word count: 3412

For peer review only - http://bmjopen.bmj.com/site/about/guidelines.xhtml

ABSTRACT

Purpose To investigate the quality of drug treatment in older patients from a physician perspective, and to describe prescribing practices by attending physicians.

Methods Descriptive pilot study of patients (≥65 years) attending a primary health care centre in Region Västra Götaland, Sweden, for a non-urgent physician consultation in January 2016. The drug treatment was assessed from a physician perspective as either appropriate or suboptimal, taking the patient's condition into account, preceded by the application of three established and extensive screening tools not to overlook *Potentially Inappropriate Medications* (PIMs) and *Potential Prescribing Omissions* (PPOs). Patients with suboptimal drug treatment were further categorized regarding priority: (i) immediate change suggested or (ii) actions suggested in the longer term. Prevalence and results of medication reviews were investigated.

Results In all, 123 patients were included (median age: 76 years; 48% female, 20% nursing home residents). When a physician perspective was applied, taking individual factors like morbidity, life expectancy, and concurrent drug treatment into account, as well as 593 partly overlapping PIMs/PPOs identified in 117 (95%) patients, a total of 45 (37%) patients had suboptimal drug treatment. Immediate handling was suggested in 13 (11%) patients, most often concerning withdrawals of propiomazine or hydroxyzine. Handling in the longer term was suggested in 32 (26%) patients, most often concerning overuse of proton pump inhibitors. Over the last year, the procedure code 'medication review' was recorded for 65 (53%) patients. In those performed during January 2016 (n=45), 23 (7%) drugs out of 309 present were acted upon, most often a dosage adjustment.

Conclusions

This pilot study shows that when a physician perspective is applied, taking priorities in the complex clinical situation into account, quality of drug treatment in primary care is in general

For peer review only - http://bmjopen.bmj.com/site/about/guidelines.xhtml

appropriate. The results may be useful in sample size considerations for future studies on prescribing practices.

Key words Aged; Inappropriate prescribing; Medication review; Pharmacoepidemiology; Prescription drugs; Primary health care.

For peer review only

For peer review only - http://bmjopen.bmj.com/site/about/guidelines.xhtml

ARTICLE SUMMARY

- This pilot study contributes current information on the quality of drug treatment in older people in primary care from the perspective of a physician, taking priorities in the complex clinical situation into account, thereby providing figures for sample size consideration in full-scale studies on prescribing practices and interventions for improved drug treatment.
- To systemize the physician assessments and ensure that important potentially inappropriate medications and potential prescribing omissions were not overlooked, the assessments were preceded by the application of extensive screening tools.
- The limited sample size and the inclusion of one primary health care centre only, reduce the external validity, and full-scale studies should include two assessors to allow estimations of inter-rater agreement.

For peer review only

BACKGROUND

The purpose of drug treatment is to increase the health of patients. However, prescribing of drugs is a challenge, particularly in older people who are sensitive to drug effects and often suffer from multiple morbidities. Numerous studies have focused on describing and improving prescribing practices in this age group.[1-5]

In order to improve the quality of drug treatment, and thereby improving patient health, some countries have incorporated medication reviews in guidelines.[6-9] Frequently, medication reviews are performed by pharmacists.[3,10] In Sweden, the National Board of Health and Welfare decided in 2012 that medication reviews lie within the professional responsibilities of the attending physician. A medication review implies that the physician reconciles the drug treatment and assesses the benefit/risk for all drugs separately and combined, to ascertain that the treatment is reasonable given the current health status.[11] Medication reviews are to be documented in the medical records by a procedure code. In the Region Västra Götaland, where this study was performed, registration of this code in primary care results in monetary compensation.

Interestingly, numerous systematic reviews have failed to demonstrate effects of specifically organized medication reviews on the outcomes mortality and hospitalizations.[12-16] Provided that low prescribing quality is a major problem in health care, this may be surprising. If, on the other hand, prescribing quality problems are less pronounced than suggested in the scientific literature,[4,5,17] the lack of patient relevant effects may be less surprising. In this context, it is important to note that studies investigating the quality of drug treatment often focus on the prevalence of *Potentially Inappropriate Medications* (PIMs) and *Potential Prescribing Omissions* (PPOs), and three in ten PIMs as well as seven in ten PPOs have been

For peer review only - http://bmjopen.bmj.com/site/about/guidelines.xhtml

shown not to be clinically relevant at the individual level.[18] Therefore, to learn more about the quality of current prescribing practices, a physician perspective, based on an overall medical assessment of the specific patient, would add important information.

Prioritizations may be an additional aspect of importance when evaluating prescribing quality concerns. As older patients often have a complex clinical situation, physicians in constraints of time may have to prioritize the most urgent needs of the patient. To the best of our knowledge, this perspective has not previously been described in the scientific literature. Further, as far as we are aware, drug treatment changes upon, and documentation of, medication reviews by the attending physicians in daily care, have not previously been described.

The aim of this pilot study, with the underlying goal to provide evidence for power calculations in full-scale studies on prescribing practices in the elderly in general, was to assess current quality of drug treatment in older patients in primary care from a physician perspective. We also wanted to describe prescribing practices by attending physicians, with focus on medication reviews as performed according to a procedure code in the medical record.

MATERIALS AND METHODS

Design and setting

A descriptive pilot study was performed in a primary health care centre belonging to the Swedish National Health Service in Region Västra Götaland. The centre serves approximately 10,000 patients and is staffed by about 10 physicians (5 general practitioners, 3 general practitioner trainees, 1 pre-registration house officer, 1 fully registered medical

For peer review only - http://bmjopen.bmj.com/site/about/guidelines.xhtml

practitioner). Two nursing homes, with approximately 100 residents, are attached to the centre.

Participants

All patients, ≥65 years of age, with a non-urgent consultation registered in a primary health care centre in January 1st to January 31st 2016, were included in the study. Thus, visits in the health care centre as well as visits in nursing homes and home visits were consecutively included if not caused by an urgent event. If a patient had >1 consultation during the study period, the first one, as well as the information available prior to this visit, was included in the study.

Data source

Data were manually extracted from electronic medical records (Asynja Visph and Journal III). For each patient, socio-demographic and health-related data were retrieved including age, sex, residence, cognition, multi-dose drug dispensing, drug treatment, and comorbidity. Regarding comorbidity, we recorded the medical conditions appearing in the Screening Tool of Older Persons´ potentially inappropriate Prescriptions (STOPP), the Screening Tool to Alert to Right Treatment (START),[19] the EU(7)-PIM list,[20] and the set of indicators of prescribing quality provided by the Swedish National Board of Health and Welfare.[21]

For each drug in the medication list, we recorded the substance name, the Anatomic Therapeutic Classification code (ATC),[22] and information as to whether the drug was prescribed for regular use or as needed. Drugs for external use were included only if having potential systemic effects. Combination drugs were counted as one drug. Two drugs including the same substance and formulation, for example two different dosages of the same drug,

For peer review only - http://bmjopen.bmj.com/site/about/guidelines.xhtml

were counted as one drug. The number of drugs that were used regularly (i.e. not for a temporary condition such as an infection) was recorded.

Presence of a procedure code in the medical records, explicitly stating that a medication review had been performed, was recorded. For patients with such a procedure code in January 2016, we recorded the actions taken and the content of the related documentation. Thus, each drug in the medication list was categorized as unchanged, withdrawn, dosage adjusted, added, or other. The changes in drug treatment were also summated at the patient level, categorized as ≥1 drugs being withdrawn, dosage adjusted, added, or other. Further, we recorded how the actions were documented in the medical records, focusing on whether the drug treatment problem was described, the goal with the treatment mentioned, and planned follow-up expressed. In order to elucidate to what extent the procedure code medication review is documented at least annually, we extended the search in the medical record for this action, if not recorded in January 2016, to the entire year preceding the physician consultation. The contents of these medication reviews were not further analysed, as the focus of the study was drug treatment as of January 2016.

Assessments

From the physician perspective

For every patient, the quality of the drug treatment was assessed at the overall level, from the perspective of a physician specialized in family medicine (NPL), with experience of assessing drug treatment quality in the elderly,[23] taking into account individual characteristics of the patient such as morbidity, life expectancy, and concurrent drug treatment. The assessor categorized each patient's drug treatment as either *appropriate* or *suboptimal*. Suboptimal treatment was further categorized according to the degree of urgency of the drug treatment

For peer review only - http://bmjopen.bmj.com/site/about/guidelines.xhtml

change: *high priority* if the suggestion was to change ≥1 drugs immediately, and *low priority* if changes could be handled in the longer term. In cases of doubt concerning the assessments, a senior specialist physician in clinical pharmacology (SMW) was consulted, both authors had a discussion, and came to a consensus decision. Information on suboptimal treatment prioritized for an immediate action was forwarded to the attending physician.

Application of screening tools prior to physician assessment

In order to systemize the physician assessments and ensure that important PIMs and PPOs were not overlooked, the overall assessment of each patient was preceded by the application of a total of 493 criteria, partly overlapping, to each patient from three established screening tools: the STOPP/START criteria version 2, consisting of 80 PIMs and 34 PPOs,[19] the EU(7)-PIM list consisting of 282 PIMs,[20] and the Swedish indicator set in which we used 97 out of 98 drug- and diagnosis-specific indicators of PIMs (n=77) as well as rational (n=20) treatments/treatment strategies;[21] one diagnosis-specific indicator was excluded because it was not applicable at the individual level. To comply with the other indicator sets, we recorded the absence of a rational treatment/treatment strategy in the Swedish indicator set as a PPO. For indicators based on kidney function, we estimated the glomerular filtration rate (eGFR) according to the Cockcroft-Gault equation.

Statistics

SPSS (IBM SPSS Statistics for Windows, Version 19.0, Armonk, NY) was used for descriptive analyses concerning characteristics of patients and drug treatment by the absence/presence of ≥1 procedure codes for medication review over the last year. In the sample size considerations for this pilot study, we considered that including all patients fulfilling our inclusion criteria during one month, anticipated to amount to about a hundred,

For peer review only - http://bmjopen.bmj.com/site/about/guidelines.xhtml

would yield useful results for the power calculations in the full-scale study, i.e. reasonably certain prevalence figures on suboptimal drug treatment as well as information on the prevalence of documented medication reviews. Values are presented as numbers (percentages) as well as mean ± standard deviation (SD) or median and interquartile range (IQR) or range where appropriate. As the Swedish regulations declare that physicians shall perform annual medication reviews for persons ≥75 years of age with ≥5 drugs in the medication list, these categories were also used.

RESULTS

A flowchart of the study population is presented in Figure 1. A total of 123 patients were included in the analyses, 59 (48%) of whom were female (Table 1). The median age was 76 years, ranging from 65 to 102 years. The number of chronic drugs was 5 (IQR: 3-7). Impaired cognition including dementia was found in 24 (20%) patients, and 24 (20%) individuals were nursing home residents. The most common medical conditions were hypertension (n=89; 72%), chronic pain (n=50; 41%), type 2 diabetes mellitus (n=39; 32%), and osteoarthritis (n=35; 28%).

Table 1 Characteristics of patients according to the presence/absence of ≥1 procedure codes indicating a performed medication review over the last year.

		≥1 procedure codes for a medication review over the last year	
		Yes (n=65)	No (n=58)
Age	Years	79 (73-84)	73 (68-79)
	≥75 years	45 (69)	19 (33)
Female sex		30 (46)	29 (50)
Impaired cognition, including dementia		16 (25)	8 (14)
Nursing home resident		15 (23)	9 (16)
Multi-dose drug dispensing		11 (17)	6 (10)

For peer review only - http://bmjopen.bmj.com/site/about/guidelines.xhtml

Morbidity	Hypertension	54 (83)	35 (60)
	Type 2 diabetes mellitus	34 (52)	5 (9)
	Chronic pain	30 (46)	20 (35)
	Gastro-oesophageal reflux disease or peptic ulcer disease	17 (26)	10 (17)
	Osteoarthritis	16 (25)	19 (33)
	Insomnia	13 (20)	13 (22)
	Cardiovascular disease	12 (19)	8 (14)
	eGFR<60 ml/min	12 (19)	6 (10)
	Dementia	11 (17)	5 (9)
	Depression	11 (17)	11 (19)
	Anxiety	9 (14)	10 (17)
Number of drugs	Total	7 (6-10)	5 (3-6)
	Regular	7 (5-8)	4 (2-6)
	As needed	1 (0-2)	1 (0-1)
	≥5 drugs	54 (83)	30 (52)

- Values are presented as median (interquartile range), or number of individuals (percentage).

eGFR, estimated Glomerular Filtration Rate.

In all, the medication lists of all patients contained 773 drugs. The most commonly prescribed drugs were paracetamol (44 patients; 34%), acetylsalicylic acid (38 patients; 30%), atorvastatin (29 patients; 24%), enalapril (29 patients; 24%), cyanocobalamin (28 patients; 23%), and metformin (27 patients; 22%).

According to the physician assessment, 45 (37%) patients had suboptimal drug treatment, that is, a change was suggested (Table 2). In 13 patients (11% of all; 29% of those with suboptimal treatment), the suggested change in drug treatment was of high priority, i.e. suggested to take place immediately, and concerned mainly withdrawal of propiomazine and hydroxyzine. In the 32 remaining cases (26% of all, 71% of those with suboptimal treatment), the suggested change was of lower priority, that is, the problem/s/ was suggested to be

For peer review only - http://bmjopen.bmj.com/site/about/guidelines.xhtml

handled in the longer term, and concerned mainly potential overtreatment with proton pump inhibitors (PPI).

Table 2 Suggested drug changes in patients with suboptimal treatment according to the physician assessment.

Priority	Suggestion	Drug (n)
High, change immediate	Withdrawal	propiomazine (n=5), hydroxyzine (n=4), diazepam (n=1), naproxen (n=1), nifedipine (n=1), orphenadrine (n=1), tramadol (n=1)
Low, reconsider in the longer term	Withdrawal, apparent indication lacking or probably inappropriate treatment	PPI (n=18), cyanocobalamin (n=2), metformin (n=2), acetylcysteine (n=1), acetylsalicylic acid (n=1), biperiden (n=1), diclofenac (n=1), dipyridamole (n=1), furosemide (n=1), laxative (n=1), potassium (n=1), quetiapine (n=1), risperidone (n=1), sotalol (n=1), zopiclone (n=1) felodipine/ carbamazepine interaction (n=1)
	Addition, absence of recommended treatment	bisphosphonate (n=1), laxatives (n=1)

- PPI, proton pump inhibitors.

For peer review only

The application of screening tools prior to the physician assessment resulted in the identification of 593 PIMs/PPOs (median: 4; IQR: 2-7), partly overlapping, in 117 (95%) patients. STOPP identified 125 PIMs in 59 (48%) patients (median: 2; IQR: 1-3), and START identified 54 PPOs in 49 (40%) patients (median: 1; IQR: 1-1). The EU(7)-PIM list identified PIMs in 58 (47%) patients (median: 1; IQR: 1-2). The Swedish indicator set identified 96 PIMs in 48 (39%) patients (median: 2; IQR: 1-3) and 226 PPOs in 105 (85%) patients (median: 2; IQR: 1-3).

A total of 65 patients (53%) had ≥1 procedure codes stating that a medication review had been performed over the last year. Among those ≥75 years of age with ≥5 drugs in the

For peer review only - http://bmjopen.bmj.com/site/about/guidelines.xhtml

medication list (n=53), 41 (77%) patients had ≥1 procedure codes registered regarding a medication review performed over the last year. The proportion of patients with suboptimal drug treatment according to the physician assessment was equally distributed between patients with and without a procedure code for a medication review performed over the last year: 24 (37%) and 21 (36%), respectively. Six of 13 patients with suboptimal treatment of high priority had a procedure code in the medical record explicitly stating that the drug treatment had been reviewed at least once over the last year. Among 32 patients with suboptimal treatment with low priority, 18 had ≥1 procedure codes regarding a medication review over the last year.

For peer review only

For 15 (33%) out of 45 patients with a procedure code for a medication review in January 2016, ≥1 actions were documented in the medical record, most often a dosage adjustment (Table 3). These actions concerned 23 (7%) drugs out of 309 present in the medication lists of these patients, and a total of 14 active substances. In all, three actions were comprehensively documented in the medical records regarding the underlying problem, the treatment goal, and the planned follow-up.

Table 3 Actions taken and contents of documentation in the medical records during documented medication reviews in January 2016 (n=45).

		Patients* n (%)	Drugs involved	Documentation in the medical records
Actions taken	Drug withdrawn	3 (7)	omeprazole (n=2)	P/G (n=1); F (n=1)
			felodipine (n=1)	P (n=1)
			sertraline (n=1)	P/G (n=1)
			zopiclone (n=1)	P/G (n=1)
	Drug dosage adjusted	10 (22)	felodipine (n=1)	P (n=1)
			insulin (n=3)	P (n=1); P/G (n=2)
			metformin (n=2)	P (n=1); P/G/F (n=1)
			metoprolol (n=1)	P (n=1)

		propiomazine (n=1)	P/G (n=1)
		repaglinide (n=1)	P/G/F (n=1)
		sertraline (n=1)	P/G (n=1)
Drug added	4 (9)	omeprazole (n=1)	P/G (n=1)
		atorvastatin (n=1)	P/F (n=1)
		enalapril (n=1)	P/G/F (n=1)
		hydrochlorothiazide (n=1)	P (n=1)
		melatonin (n=1)	P/G (n=1)
		paracetamol (n=1)	P (n=1)
Other	2 (4)	enalapril (changed to other brand because of suspected side effects) (n=1)	P/G (n=1)
		metformin (compliance discussed) (n=1)	P (n=1)

- F, planned follow-up; G, treatment goal; N/A, not applicable; P, drug treatment problem

* ≥1 actions could be recorded for a single patient.

DISCUSSION

Main findings

In this study, we show that more than six in ten patients in primary care had appropriate drug treatment, when assessed from an overall perspective by a physician, taking morbidity, life expectancy, and concurrent drug treatment into account. Only one in ten patients had suboptimal drug treatment where an immediate change was suggested to be prioritized, mostly concerning a withdrawal of sedatives/anxiolytics. For one in four patients, the suboptimal treatment was suggested to be of lower priority, predominantly concerning the withdrawal of a PPI.

According to the procedure codes in the medical records, primary care physicians documented medication reviews in about half of all older patients at least annually, with focus on those of old age with extensive comorbidities and long medication lists. For one in three patients, the medication review resulted in an active change of treatment, most often a dosage adjustment.

For peer review only - http://bmjopen.bmj.com/site/about/guidelines.xhtml

Strengths and limitations

To the best of our knowledge this is the first study to provide information on the quality of drug treatment in older people in primary care from the perspective of a physician, taking priorities in the complex clinical situation into account. Thereby, our pilot study contributes valuable information for sample size consideration in future studies on prescribing practices and interventions for improved drug treatment. Another strength is that the physician assessment was preceded by extensive screening for PIMs and PPOs, thus minimizing the risk of overlooking important aspects regarding the prescribing quality. Indeed, only one in twenty patients did not have any PIMs/PPOs, indicating that our screening tools captured potentially suboptimal treatment to a greater extent than many other studies.[4,5,17] An additional strength is the fact that only one patient was excluded, an important aspect for generalizability.

Limitations of this study include the sample size and the fact that data were obtained from one health care centre only. This may reduce the external validity. However, a limited sample size may be justified in a pilot study; a full-scale study, sized on the basis of these pilot results, has been initiated. Another limitation is that the physician assessments were performed by one person only. However, this physician had relevant expertise as well as previous experience of scientifically assessing pharmacotherapy in the elderly.[23] In addition, ambiguities in the assessments were discussed with a senior clinical pharmacologist. Nevertheless, full-scale studies should preferably include two assessors and consensus discussions to provide information on inter-rater agreement. Another limitation is that the study was restricted to information in the medical records from primary care. Indeed, the assessments were based on available data in routine care, carrying a risk that the information

For peer review only - http://bmjopen.bmj.com/site/about/guidelines.xhtml

about the medical history, diagnoses, examinations, and laboratory tests had not been documented or appropriately coded in the medical records. Yet, this data source is more comprehensive than drug registers, either alone or linked to other registers, which are frequently used in studies on prescribing practices.[24]

Interpretation

The apparent discrepancy between the prevalence of suboptimal drug treatment according to the physician assessment and the screening tools illustrates the importance of taking future research on prescribing practices a step further to increase the medical relevance. Our results suggest that the limitations regarding the concurrent validity of general screening tools in hip fracture patients[18,25-27] may also apply to older patients in general in primary care. Indeed, although the positive predictive value for several indicator sets has been reported to be acceptable when determined separately,[25,26] an overall assessment by a physician may provide lower figures.

The low prevalence of suboptimal drug treatment where an immediate change was suggested contributes to the understanding of the medical practice in primary care. In fact, the level of priority, according to our definition, may represent a balance between feasibility and expected patient harm; changes that can be achieved with reasonable efforts in relation to the anticipated patient benefit will get a higher priority than the opposite. Indeed, the changes suggested by the assessing physician predominantly concerned sedatives/anxiolytics and PPIs, withdrawals of which may not easily be achieved once they have been initiated.[28,29] The high prevalence of PPI without an underlying indication is in agreement with a recent population-based register-based study where no underlying disease- or a drug-related reason could be identified for four in ten patients on long-term treatment with PPI.[30] Our pilot

For peer review only - http://bmjopen.bmj.com/site/about/guidelines.xhtml

findings also contributes to the understanding of the limited implementation of recommendations for drug changes after medication reviews by third parties.[31]

Interestingly, the proportion of patients with suboptimal drug treatment was similar for patients with and without a procedure code explicitly stating that a medication review had been performed over the last year. However, patients with such a procedure code had more extensive comorbidity, also illustrated by the higher number of drugs in the medication list, which has been shown to be an acceptable surrogate of burden of disease.[32] Thus, the treatment in multimorbid patients can be expected to be more complex, and it is reassuring that drug treatment quality is acceptable to the same extent also in these patients. Although this pilot study does not allow conclusions, one may speculate that performed medication reviews may have contributed to appropriate drug treatment in multimorbid patients requiring complex treatment considerations.

Patients as defined in the Swedish regulations to receive a medication review at least annually, that is, patients ≥75 years of age with ≥5 drugs in the medication list, had this procedure code documented to a large extent. The adherence to this regulation, despite the absence of an established association between age and drug treatment quality,[18] and the limitations of cut-offs of number of drugs in the medication list to reflect quality of drug treatment,[33] may illustrate that monetary compensations can direct health care efforts to some extent. However, the evidence for effects of pay for performance policies in improving quality of care and health outcomes is limited.[34] Indeed, a recent study, investigating the impact of pay for performance regarding the procedure code ‘medication review’ found no apparent association between this code and the prevalence of PIMs, although the number of patients with such a code increased.[35]

For peer review only - http://bmjopen.bmj.com/site/about/guidelines.xhtml

The actions performed within the documented medication reviews were quite few, as only one out of 14 drugs were acted upon. This finding may give the impression that medication reviews by the attending physician is a minor effort. However, as a medication review implies a medical assessment, ascertaining that the drug treatment is reasonable given the current health status, the decision not to change a drug may be just as important. Moreover, the basic part of a medication review, the medication reconciliation, was not captured in the present study.

Our finding that the problem underlying the treatment action was documented in the medical records in all but one case is positive. Indeed, such information is important in transitions of care. Nevertheless, there may be potentials for improvements as the goal of the drug treatment change was only stated in about half of the cases, and the planned follow-up in a minority.

In conclusion, the results of this pilot study suggest that current prescribing practices in health care may be quite good, potentially due to efforts over the last decades for rational use of medicines, for example easily available information on recommended drugs,[36,37] computerized decision support,[38,39] and indicators of prescribing quality for benchmarking and educational purposes.[21,40] Indeed, although the latter may reflect quality of drug treatment to a limited extent, they may be valuable in particular for junior physicians, as they reflect expert opinions on current best practice for an average older patient. We also want to emphasize that, although our findings indicate that drug treatment in older people in primary care is in general appropriate, endeavors for improvements are always desirable. Our results

For peer review only - http://bmjopen.bmj.com/site/about/guidelines.xhtml

may be useful in sample size considerations for future non-randomized and randomized studies, focusing to factors associated with prescribing practices as well and interventions.

For peer review only

For peer review only - http://bmjopen.bmj.com/site/about/guidelines.xhtml

STATEMENTS

Author contributorship: N.P.L. contributed to study design, acquisition of data, analysis and interpretation of results. S.M.W. contributed to study design, analysis and interpretation of results. Both authors drafted and revised the article, and approved the final manuscript.

Conflict of Interest: The authors declare that they have no conflict of interest.

Funding: This work was supported by the Swedish Research Council; LUA/ALF grants; and the Department of Research and Development in Södra Älvsborg.

Patient consent: Not required.

Ethical approval: The study was approved by the Regional Ethical Review Board in Gothenburg, Sweden (DRN: 1046-15). Informed consent was waived in the approval.

Data sharing: The datasets generated and analysed during the current study are not publicly available due to Swedish data protection laws. The data can be shared with authorized persons after approved application from the Regional Ethical Review Board in Gothenburg (https://www.epn.se/en/start/the-organisation/).

For peer review only

REFERENCES

1. Alldred DP, Kennedy MC, Hughes C, et al. Interventions to optimise prescribing for older people in care homes. *Cochrane Database Syst Rev* 2016;**2**:Cd009095.

2. Gillaizeau F, Chan E, Trinquart L, et al. Computerized advice on drug dosage to improve prescribing practice. *Cochrane Database Syst Rev* 2013(11):CD002894.

3. Patterson SM, Cadogan CA, Kerse N, et al. Interventions to improve the appropriate use of polypharmacy for older people. *Cochrane Database Syst Rev* 2014;**10**.

4. Tommelein E, Mehuys E, Petrovic M, et al. Potentially inappropriate prescribing in community-dwelling older people across Europe: a systematic literature review. *Eur J Clin Pharmacol* 2015;**71**(12):1415-27.

5. Guaraldo L, Cano FG, Damasceno GS, et al. Inappropriate medication use among the elderly: a systematic review of administrative databases. *BMC geriatrics* 2011;**11**:79.

6. Clyne W, Blenkinsopp A, Seal R. A guide to medication review. Secondary A guide to medication review [Web Page] 2008. http://www.cff.org.br/userfiles/52%20-%20CLYNE%20W%20A%20guide%20to%20medication%20review%202008.pdf.

7. Marengoni A, Onder G. Guidelines, polypharmacy, and drug-drug interactions in patients with multimorbidity. *Bmj* 2015;**350**:h1059.

8. Zhan C, Sangl J, Bierman AS, et al. Potentially inappropriate medication use in the community-dwelling elderly: findings from the 1996 Medical Expenditure Panel Survey. *Jama* 2001;**286**(22):2823.

9. The Federal Union of German Associations of Pharmacists. Grundsatzpapier zur Medikationsanalyse und zum Medikationsmanagement [Medication analysis and Medication management]. Secondary Grundsatzpapier zur Medikationsanalyse und zum Medikationsmanagement [Medication analysis and Medication management] [Web Page] 2014.

For peer review only - http://bmjopen.bmj.com/site/about/guidelines.xhtml

http://www.abda.de/fileadmin/assets/Medikationsmanagement/Grundsatzpapier_MA_MM_GBAM.pdf.

10. Holland R, Desborough J, Goodyer L, et al. Does pharmacist□led medication review help to reduce hospital admissions and deaths in older people? A systematic review and meta□analysis. *British Journal of Clinical Pharmacology* 2008;**65**(3):303-16.

11. The National Board of Health and Welfare. Senaste version av SOSFS 2000:1. Socialstyrelsens föreskrifter och allmänna råd om läkemedelshantering i hälso- och sjukvården [Medication reviews]. Secondary Senaste version av SOSFS 2000:1. Socialstyrelsens föreskrifter och allmänna råd om läkemedelshantering i hälso- och sjukvården [Medication reviews] 2016. https://lakemedelsverket.se/upload/om-lakemedelsverket/NLS/Socialstyrelsen.se.pdf.

12. Christensen M, Lundh A. Medication review in hospitalised patients to reduce morbidity and mortality. *Cochrane Database Syst Rev* 2016;**2**:Cd008986.

13. Hohl CM, Wickham ME, Sobolev B, et al. The effect of early in-hospital medication review on health outcomes: a systematic review. *Br J Clin Pharmacol* 2015;**80**(1):51-61.

14. Wallerstedt SM, Kindblom JM, Nylén K, et al. Medication reviews for nursing home residents to reduce mortality and hospitalization: systematic review and meta-analysis. *British Journal of Clinical Pharmacology* 2014;**78**(3):488.

15. Lehnbom EC, Stewart MJ, Manias E, et al. Impact of medication reconciliation and review on clinical outcomes. *The Annals of pharmacotherapy* 2014;**48**(10):1298-312.

16. Huiskes VJ, Burger DM, van den Ende CH, et al. Effectiveness of medication review: a systematic review and meta-analysis of randomized controlled trials. *BMC family practice* 2017;**18**(1):5.

For peer review only - http://bmjopen.bmj.com/site/about/guidelines.xhtml

17. Hill-Taylor B, Sketris I, Hayden J, et al. Application of the STOPP/START criteria: a systematic review of the prevalence of potentially inappropriate prescribing in older adults, and evidence of clinical, humanistic and economic impact. *J Clin Pharm Ther* 2013;**38**(5):360.

18. Lönnbro J, Wallerstedt SM. Clinical relevance of the STOPP/START criteria in hip fracture patients. *Eur J Clin Pharmacol* 2017;**73**(4):499-505.

19. O'Mahony D, O'Sullivan D, Byrne S, et al. STOPP/START criteria for potentially inappropriate prescribing in older people: version 2. *Age Ageing* 2015;**44**(2):213.

20. Renom-Guiteras A, Meyer G, Thürmann PA. The EU(7)-PIM list: a list of potentially inappropriate medications for older people consented by experts from seven European countries. *European Journal of Clinical Pharmacology* 2015;**71**(7):861.

21. Fastbom J, Johnell K. National indicators for quality of drug therapy in older persons: the Swedish experience from the first 10 years. *Drugs Aging* 2015;**32**(3):189.

22. WHO Collaborating Centre for Drug Statistics Methodology. Guidelines for ATC classification and DDD assignment 2013. Secondary WHO Collaborating Centre for Drug Statistics Methodology. Guidelines for ATC classification and DDD assignment 2013 2016/07/05/08:38:58 2012. http://www.whocc.no/filearchive/publications/1_2013guidelines.pdf.

23. Parodi López N, Villán Villán YF, Granados Menéndez MI, et al. Prescripción potencialmente inapropiada en mayores de 65 años en un centro de salud de atención primaria [Potentially inappropriate prescribing in patients over 65 years-old in a primary care health centre]. *Aten Primaria* 2014;**46**(6):290-7.

24. Wallerstedt SM, Wettermark B, Hoffmann M. The First Decade with the Swedish Prescribed Drug Register - A Systematic Review of the Output in the Scientific Literature. *Basic Clin Pharmacol Toxicol* 2016;**119**(5):464-69.

For peer review only - http://bmjopen.bmj.com/site/about/guidelines.xhtml

25. Wallerstedt SM, Belfrage B, Koldestam A, et al. [Many patients with suboptimal drug treatment are missed. Sensitivity, specificity and prediction value of drug-specific quality indicators for the elderly]. *Lakartidningen* 2016;**113**:DSU4.

26. Wallerstedt SM, Belfrage B, Fastbom J. Association between drug-specific indicators of prescribing quality and quality of drug treatment: a validation study. *Pharmacoepidemiology and Drug Safety* 2015;**24**:906-14.

27. Dalleur O, Boland B, De Groot A, et al. Detection of potentially inappropriate prescribing in the very old: cross-sectional analysis of the data from the BELFRAIL observational cohort study. *BMC Geriatr* 2015;**15**:156.

28. Hedenrud TM, Svensson SA, Wallerstedt SM. "Psychiatry is not a science like others" - a focus group study on psychotropic prescribing in primary care. *BMC Fam Pract* 2013;**14**:115.

29. Lodrup AB, Reimer C, Bytzer P. Systematic review: symptoms of rebound acid hypersecretion following proton pump inhibitor treatment. *Scand J Gastroenterol* 2013;**48**(5):515-22.

30. Wallerstedt SM, Fastbom J, Linke J, et al. Long-term use of proton pump inhibitors and prevalence of disease- and drug-related reasons for gastroprotection-a cross-sectional population-based study. *Pharmacoepidemiol Drug Saf* 2016;**16**(1):9-16.

31. Kwint HF, Bermingham L, Faber A, et al. The relationship between the extent of collaboration of general practitioners and pharmacists and the implementation of recommendations arising from medication review: a systematic review. *Drugs Aging* 2013;**30**(2):91-102.

32. Brilleman SL, Salisbury C. Comparing measures of multimorbidity to predict outcomes in primary care: a cross sectional study. *Fam Pract* 2013;**30**(2):172-8.

For peer review only - http://bmjopen.bmj.com/site/about/guidelines.xhtml

33. Belfrage B, Koldestam A, Sjöberg C, et al. Number of drugs in the medication list as an indicator of prescribing quality: a validation study of polypharmacy indicators in older hip fracture patients. *European Journal of Clinical Pharmacology* 2015;**71**(3):363.

34. Rashidian A, Omidvari AH, Vali Y, et al. Pharmaceutical policies: effects of financial incentives for prescribers. *Cochrane Database Syst Rev* 2015(8):Cd006731.

35. Ödesjö H, Anell A, Boman A, et al. Pay for performance associated with increased volume of medication reviews but not with less inappropriate use of medications among the elderly – an observational study. *Scand J Prim Health Care* 2017;**35**(3):271-78.

36. Bjorkhem-Bergman L, Andersen-Karlsson E, Laing R, et al. Interface management of pharmacotherapy. Joint hospital and primary care drug recommendations. *Eur J Clin Pharmacol* 2013;**69 Suppl 1**:73-8.

37. Axelsson MA, Spetz M, Mellen A, et al. Use of and attitudes towards the prescribing guidelines booklet in primary health care doctors. *BMC Clin Pharmacol* 2008;**8**:8.

38. Hellden A, Al-Aieshy F, Bastholm-Rahmner P, et al. Development of a computerised decisions support system for renal risk drugs targeting primary healthcare. *BMJ Open* 2015;**5**(7):e006775.

39. Bottiger Y, Laine K, Andersson ML, et al. SFINX-a drug-drug interaction database designed for clinical decision support systems. *Eur J Clin Pharmacol* 2009;**65**(6):627-33.

40. Rognstad S, Brekke M, Fetveit A, et al. Prescription peer academic detailing to reduce inappropriate prescribing for older patients: a cluster randomised controlled trial. *Br J Gen Pract* 2013;**63**(613):e554-62.

For peer review only - http://bmjopen.bmj.com/site/about/guidelines.xhtml

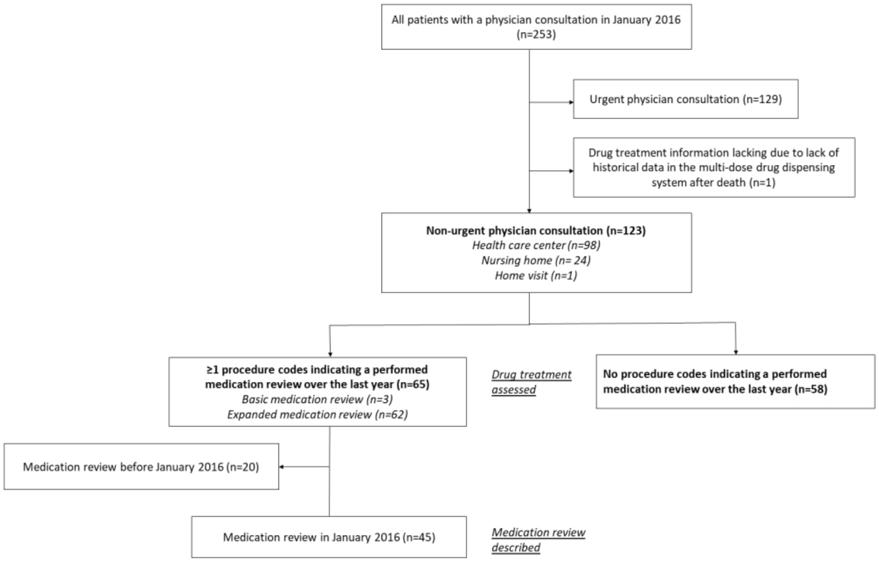

Figure 1 Flowchart of the study population

489x275mm (96 x 96 DPI)

BMJ Open

Quality of prescribing in older people from a broad family physician perspective: A descriptive pilot study

Journal:	*BMJ Open*
Manuscript ID	bmjopen-2018-027290.R1
Article Type:	Research
Date Submitted by the Author:	20-Dec-2018
Complete List of Authors:	Parodi Lopez, Naldy; Närhälsan Vårgårda Health Centre; Sahlgrenska Academy, University of Gothenburg, Department of Pharmacology<br>Wallerstedt, Susanna; Sahlgrenska Academy, University of Gothenburg, Department of Pharmacology; Sahlgrenska universitetssjukhuset, HTA-centrum
**Primary Subject Heading**:	General practice / Family practice
Secondary Subject Heading:	Pharmacology and therapeutics
Keywords:	Aged, Inappropriate prescribing, Medication review, Pharmacoepidemiology, Prescription drugs, Primary health care

SCHOLARONE™
Manuscripts

Quality of prescribing in older people from a broad family physician perspective: A descriptive pilot study

Naldy Parodi Lopez[1,2], Susanna M. Wallerstedt[2,3]

[1]Närhälsan Vårgårda Health Centre, Sweden

[2]Department of Pharmacology, Sahlgrenska Academy, University of Gothenburg, Gothenburg, Sweden

[3]HTA-centrum, Sahlgrenska University Hospital, Gothenburg, Sweden

Corresponding author:

Naldy Parodi Lopez, Närhälsan Vårgårda Health Centre, Centrumgatan 2, 44731, Vårgårda, Sweden. Tel.:+46722600348; E-mail: naldy.parodi.lopez@vgregion.se

Word count: 3915

For peer review only - http://bmjopen.bmj.com/site/about/guidelines.xhtml

ABSTRACT

Objectives To investigate the quality of drug treatment in older people from a broad family physician perspective, and to provide evidence for power calculations in full-scale studies on prescribing quality.

Design Descriptive, retrospective pilot study.

Setting A primary health care centre in Sweden.

Participants 123 consecutive patients, ≥65 years, with a non-urgent physician consultation in January 2016.

Measures The drug treatment was assessed by a physician as either appropriate or suboptimal, taking individual factors like morbidity, life expectancy, and concurrent drug treatment into account, and preceded by the application of 493 criteria from three screening tools for *Potentially Inappropriate Medications* (PIMs) and *Potential Prescribing Omissions* (PPOs). Suboptimal drug treatment was further categorized regarding priority: (i) immediate change suggested or (ii) actions suggested in the longer term. Prevalence of the procedure code 'medication review' and the results thereof were also recorded.

Results Median age: 76 years; 48% female. When a broad family physician perspective was applied, and 593 PIMs/PPOs identified in 117 (95%) patients considered, 45 (37%) patients had suboptimal drug treatment. Immediate handling was suggested in 13 (11%) patients, most often concerning withdrawals of drugs for anxiety and insomnia. Handling in the longer term was suggested in 32 (26%) patients, most often concerning overuse of proton pump inhibitors. Over the last year, the procedure code 'medication review' was recorded for 65 (53%) patients. In medication reviews recorded during January 2016 (n=45), 23 (7%) drugs out of 309 were acted upon, most often a dosage adjustment.

Conclusions

For peer review only - http://bmjopen.bmj.com/site/about/guidelines.xhtml

This pilot study shows that when a broad family physician perspective is applied, taking individual factors and medical priorities in the complex clinical situation into account, drug treatment in primary care is appropriate for the majority of older patients. The results may be useful in sample size considerations for future studies on prescribing practices.

Key words Aged; Inappropriate prescribing; Medication review; Pharmacoepidemiology; Prescription drugs; Primary health care.

For peer review only

For peer review only - http://bmjopen.bmj.com/site/about/guidelines.xhtml

ARTICLE SUMMARY

- This pilot study contributes current and clinically relevant information on the quality of drug treatment in older people from a broad family physician perspective, taking patient factors and medical priorities in the complex clinical situation into account, thereby providing figures for sample size consideration in full-scale studies on prescribing quality.
- To systemize the physician assessments and ensure that important potentially inappropriate medications and potential prescribing omissions were not overlooked, the assessments were preceded by the application of extensive screening tools.
- The limited sample size and the inclusion of one primary health care centre only, reduce the external validity, and full-scale studies should include two assessors to allow estimations of inter-rater agreement.

For peer review only

For peer review only - http://bmjopen.bmj.com/site/about/guidelines.xhtml

BACKGROUND

The purpose of drug treatment is to increase the health of patients. However, prescribing of drugs is a challenge, particularly in older people who are sensitive to drug effects and often suffer from multiple morbidities. Numerous studies have focused on describing and improving prescribing practices in this age group.[1-5]

In order to improve the quality of drug treatment, and thereby improving patient health, some countries have incorporated medication reviews in guidelines.[6-9] Frequently, medication reviews are performed by pharmacists.[3,10] In Sweden, the National Board of Health and Welfare decided in 2012 that medication reviews lie within the professional responsibilities of the physician; the only profession with a full licence to prescribe. According to national regulations, a medication review implies that the physician reconciles the drug treatment and assesses the benefit/risk for all drugs separately and combined, to ascertain that the treatment is reasonable given the current health status.[11] Medication reviews are to be documented in the medical records by a procedure code. In the Region Västra Götaland, where this study was performed, registration of this code in primary care results in monetary compensation.

Interestingly, numerous systematic reviews have failed to demonstrate effects of specifically organized medication reviews on the outcomes mortality and hospitalizations.[12-16] Provided that inappropriate prescribing is a major problem in health care,[4,5,17] this may be surprising. If, on the other hand, prescribing quality problems are less pronounced than the scientific literature may suggest, the lack of patient relevant effects may be less surprising. In this context, it is important to note that studies investigating the quality of drug treatment often focus on the prevalence of *Potentially Inappropriate Medications* (PIMs) and *Potential Prescribing Omissions* (PPOs), and three in ten PIMs as well as seven in ten PPOs have been

For peer review only - http://bmjopen.bmj.com/site/about/guidelines.xhtml

shown not to be clinically relevant at the individual level.[18] Therefore, to learn more about the quality of current prescribing practices, a broader physician approach, based on an overall medical assessment of the information available for a specific patient, would add important information.

Prioritizations may be an additional aspect of importance when evaluating prescribing quality concerns. As older people often have a complex clinical situation, physicians in constraints of time may have to prioritize the most urgent needs of the patient. To the best of our knowledge, this perspective has not previously been described in the scientific literature. Further, as far as we are aware, drug treatment changes upon, and documentation of, medication reviews by the attending physicians in daily care, have not previously been described.

The aim of this pilot study, with the underlying goal to provide evidence for power calculations in full-scale studies on prescribing quality, was to investigate the quality of drug treatment in older people from a broad family physician perspective, taking individual factors and medical priorities in the complex clinical situation into account. We also wanted to describe prescribing practices by physicians in primary care, with focus on medication reviews as performed according to a procedure code in the medical record.

MATERIALS AND METHODS

Design and setting

A descriptive pilot study was performed in a primary health care centre belonging to the Swedish National Health Service in Region Västra Götaland. The centre serves approximately 10,000 patients and is staffed by about 10 physicians (5 specialists in family

For peer review only - http://bmjopen.bmj.com/site/about/guidelines.xhtml

medicine, 3 residents in family medicine, 2 licensed physicians without specialist competence, and one intern). Two nursing homes, with approximately 100 residents, are attached to the centre.

Participants

All patients, ≥65 years of age, with a non-urgent consultation registered in a primary health care centre in January 1st to January 31st 2016, were included in the study. Thus, visits in the health care centre as well as visits in nursing homes and home visits were consecutively included if not caused by an urgent event. If a patient had >1 consultation during the study period, the first one, as well as the information available prior to this visit, was included in the study.

Data source

Data were retrospectively and manually extracted from electronic medical records (Asynja Visph and Journal III). The centre physicians were not informed beforehand that their work would be monitored the month in question. For each patient, socio-demographic and health-related data were retrieved including age, sex, residence, cognition, multi-dose drug dispensing (machine-dispensed unit bags with drugs that should be ingested concomitantly, for patients who have difficulties in handling their medications), drug treatment, and morbidities. Regarding morbidities, we recorded the presence of medical conditions appearing in the Screening Tool of Older Persons´ potentially inappropriate Prescriptions (STOPP), the Screening Tool to Alert to Right Treatment (START),[19] the EU(7)-PIM list,[20] and the set of indicators of prescribing quality provided by the Swedish National Board of Health and Welfare.[21]

For peer review only - http://bmjopen.bmj.com/site/about/guidelines.xhtml

For each drug in the current medication list, at the end of the visit according to the medical records including electronic prescriptions with detailed information on prescribed products and doses, we recorded the substance name, the Anatomic Therapeutic Classification code (ATC),[22] and information as to whether the drug was prescribed for regular use or as needed. Drugs for external use were included only if having potential systemic effects. Combination drugs were counted as one drug. Two drugs including the same substance and formulation, for example two different dosages of the same drug, were counted as one drug. The number of drugs that were used regularly (i.e. not for a temporary condition such as an infection) was recorded.

We recorded if a procedure code, explicitly stating that a medication review had been performed, was present or absent in the medical records of the physician consultation in January 2016. If present, we recorded the actions taken and the content of the related documentation, based on the information available in the medical records and the electronic prescriptions. Thus, each drug in the medication list was categorized as unchanged, withdrawn, dosage adjusted, added, or other. The changes in drug treatment were also summated at the patient level, categorized as ≥1 drugs being withdrawn, dosage adjusted, added, or other. Further, we recorded how the actions were documented in the medical records, focusing on whether the drug treatment problem was described, the goal with the treatment mentioned, and planned follow-up expressed. In order to elucidate to what extent the procedure code medication review is documented at least annually, we extended the search in the medical record for this action, if not recorded in January 2016, to the entire year preceding the physician consultation. The contents of these medication reviews were not further analysed, as the focus of the study was drug treatment as of January 2016.

For peer review only - http://bmjopen.bmj.com/site/about/guidelines.xhtml

Assessments

From a broad family physician perspective

For every patient, the quality of the drug treatment was assessed at the overall level, from the perspective of a physician specialized in family medicine (NPL), with experience of assessing drug treatment quality in older people,[23] taking into account individual characteristics of the patient such as morbidity, life expectancy, and concurrent drug treatment. The assessments were based on the information in the electronic medical records. The assessor categorized each patient's drug treatment as either *appropriate* or *suboptimal*. Suboptimal treatment was further categorized according to the suggested handling of the drug treatment change: *high priority* if the suggestion was to change ≥1 drugs immediately, and *low priority* if changes could be handled in the longer term. In cases of doubt concerning the assessments, a senior specialist physician in clinical pharmacology (SMW) was consulted, both authors had a discussion, and came to a consensus decision. Information on suboptimal treatment prioritized for an immediate action was forwarded to the physician who attended the patient.

Application of screening tools prior to physician assessment

In order to systemize the physician assessments and ensure that important PIMs and PPOs were not overlooked, the overall assessment of each patient was preceded by the manual application of a total of 493 criteria from three established screening tools: the STOPP/START criteria version 2, consisting of 80 PIMs and 34 PPOs,[19] the EU(7)-PIM list consisting of 282 PIMs,[20] and the Swedish indicator set in which we used 97 out of 98 drug- and diagnosis-specific indicators of PIMs (n=77) as well as rational (n=20) treatments/treatment strategies;[21] one diagnosis-specific indicator was excluded because it was not applicable at the individual level. To comply with the other indicator sets, we

For peer review only - http://bmjopen.bmj.com/site/about/guidelines.xhtml

recorded the absence of a rational treatment/treatment strategy in the Swedish indicator set as a PPO. The sets partly overlapped as, for example, potential overuse of proton pump inhibitors and long-acting benzodiazepines were alerted in all sets. Renal function, reflected in the estimated glomerular filtration rate (eGFR), was extracted from the medical records. For patients where an eGFR was not provided in this source, we calculated creatinine clearance according to the Cockcroft-Gault equation. The assessing physician had a hard copy with all criteria, and all detected PIMs/PPOs were marked for each patient. The assessor was also allowed to identify inappropriate drug treatment not covered by the screening tools.

Statistics

SPSS (IBM SPSS Statistics for Windows, Version 19.0, Armonk, NY) was used for descriptive analyses concerning characteristics of patients and drug treatment by the absence/presence of ≥1 procedure codes for medication review over the last year. In the sample size considerations for this pilot study, we considered that including all patients fulfilling our inclusion criteria during one month, anticipated to amount to about a hundred, would yield useful results for the power calculations in the full-scale study, i.e. reasonably certain prevalence figures on suboptimal drug treatment as well as information on the prevalence of documented medication reviews. Values are presented as numbers (percentages) as well as mean ± standard deviation (SD) or median and interquartile range (IQR) or range where appropriate. As the Swedish regulations declare that physicians shall perform annual medication reviews for persons ≥75 years of age with ≥5 drugs in the medication list, these categories were also used.

Patient and public involvement

For peer review only - http://bmjopen.bmj.com/site/about/guidelines.xhtml

This research was done without patient involvement. Patients were not invited to comment on the study design and were not consulted to develop patient relevant outcomes or interpret the results. Patients were not invited to contribute to the writing or editing of this document for readability or accuracy.

RESULTS

A flowchart of the study population is presented in Figure 1. A total of 123 patients were included in the analyses, 59 (48%) of whom were female (Table 1). The median age was 76 years, ranging from 65 to 102 years. The number of chronic drugs was 5 (IQR: 3-7). Impaired cognition including dementia was found in 24 (20%) patients, and 24 (20%) individuals were nursing home residents. The most common medical conditions were hypertension (n=89; 72%), chronic pain (n=50; 41%), type 2 diabetes mellitus (n=39; 32%), and osteoarthritis (n=35; 28%).

Table 1 Characteristics of patients according to the presence/absence of ≥1 procedure codes indicating a performed medication review over the last year.

		≥1 procedure codes for a medication review over the last year	
		Yes (n=65)	No (n=58)
Age	Years	79 (73-84)	73 (68-79)
	≥75 years	45 (69)	19 (33)
Female sex		30 (46)	29 (50)
Impaired cognition, including dementia		16 (25)	8 (14)
Nursing home resident		15 (23)	9 (16)
Multi-dose drug dispensing		11 (17)	6 (10)
Common morbidities in the sample	Hypertension	54 (83)	35 (60)
	Type 2 diabetes mellitus	34 (52)	5 (9)
	Chronic pain	30 (46)	20 (35)
	Gastro-oesophageal reflux	17 (26)	10 (17)

For peer review only - http://bmjopen.bmj.com/site/about/guidelines.xhtml

	disease or peptic ulcer disease		
	Osteoarthritis	16 (25)	19 (33)
	Insomnia	13 (20)	13 (22)
	Cardiovascular disease	12 (19)	8 (14)
	eGFR<60 ml/min	12 (19)	6 (10)
	Dementia	11 (17)	5 (9)
	Depression	11 (17)	11 (19)
	Anxiety	9 (14)	10 (17)
Number of drugs post-visit	Total	7 (6-10)	5 (3-6)
	Regular	7 (5-8)	4 (2-6)
	As needed	1 (0-2)	1 (0-1)
	≥5 drugs	54 (83)	30 (52)

- Values are presented as median (interquartile range), or number of individuals (percentage).

eGFR, estimated Glomerular Filtration Rate.

In all, the medication lists of all patients contained 773 drugs. The most commonly prescribed drugs were paracetamol (44 patients; 34%), acetylsalicylic acid (38 patients; 30%), atorvastatin (29 patients; 24%), enalapril (29 patients; 24%), cyanocobalamin (28 patients; 23%), and metformin (27 patients; 22%).

According to the physician assessment, 78 (63%) patients had *appropriate* treatment and 45 (37%) patients had *suboptimal* drug treatment, that is, a change was suggested (Table 2). In 13 patients (11% of all; 29% of those with suboptimal treatment), the suggested change in drug treatment was of high priority, i.e. suggested to take place immediately, and concerned mainly withdrawal of propiomazine (an atypical antipsychotic agent with sedative properties used for insomnia) and hydroxyzine (a first-generation antihistamine primarily used for anxiety). In the 32 remaining cases (26% of all, 71% of those with suboptimal treatment), the suggested change was of lower priority, that is, the problem/s/ was suggested to be handled in

For peer review only

For peer review only - http://bmjopen.bmj.com/site/about/guidelines.xhtml

the longer term, and concerned mainly potential overtreatment with proton pump inhibitors (PPI).

Table 2 Suggested drug changes in patients with suboptimal treatment according to the physician assessment.

Priority	Suggestion	Drug (n)
High, change immediate	Withdrawal	propiomazine (n=5), hydroxyzine (n=4), diazepam (n=1), naproxen (n=1), nifedipine (n=1), orphenadrine (n=1), tramadol (n=1)
Low, reconsider in the longer term	Withdrawal, apparent indication lacking or probably inappropriate treatment	PPI (n=18), cyanocobalamin (n=2), metformin (n=2), acetylcysteine (n=1), acetylsalicylic acid (n=1), biperiden (n=1), diclofenac (n=1), dipyridamole (n=1), furosemide (n=1), laxative (n=1), potassium (n=1), quetiapine (n=1), risperidone (n=1), sotalol (n=1), zopiclone (n=1) felodipine/ carbamazepine interaction (n=1)
	Addition, absence of recommended treatment	bisphosphonate (n=1), laxatives (n=1)

- PPI, proton pump inhibitors.

The application of screening tools prior to the physician assessment resulted in the identification of 593 PIMs/PPOs (median: 4; IQR: 2-7), partly overlapping, in 117 (95%) patients. STOPP identified 125 PIMs in 59 (48%) patients (median: 2; IQR: 1-3), and START identified 54 PPOs in 49 (40%) patients (median: 1; IQR: 1-1). The EU(7)-PIM list identified PIMs in 58 (47%) patients (median: 1; IQR: 1-2). The Swedish indicator set identified 96 PIMs in 48 (39%) patients (median: 2; IQR: 1-3) and 226 PPOs in 105 (85%) patients (median: 2; IQR: 1-3).

A total of 65 patients (53%) had ≥1 procedure codes stating that a medication review had been performed over the last year. Among those ≥75 years of age with ≥5 drugs in the

For peer review only - http://bmjopen.bmj.com/site/about/guidelines.xhtml

medication list (n=53), 41 (77%) patients had ≥1 procedure codes registered regarding a medication review performed over the last year. The proportion of patients with suboptimal drug treatment according to the physician assessment was equally distributed between patients with and without a procedure code for a medication review performed over the last year: 24 (37%) and 21 (36%), respectively. Six of 13 patients with suboptimal treatment of high priority had a procedure code in the medical record explicitly stating that the drug treatment had been reviewed at least once over the last year. Among 32 patients with suboptimal treatment with low priority, 18 had ≥1 procedure codes regarding a medication review over the last year.

For 15 (33%) out of 45 patients with a procedure code for a medication review in January 2016, ≥1 actions were documented in the medical record, most often a dosage adjustment (Table 3). These actions concerned 23 (7%) drugs out of 309 present in the medication lists of these patients, and a total of 14 active substances. In all, three actions were comprehensively documented in the medical records regarding the underlying problem, the treatment goal, and the planned follow-up. There were no changes in drug treatment due to reduced renal function.

Table 3 Actions taken and contents of documentation in the medical records during documented medication reviews in January 2016 (n=45).

		Patients* n (%)	Drugs involved	Documentation in the medical records
Actions taken	Drug withdrawn	3 (7)	omeprazole (n=2)	P/G (n=1); F (n=1)
			felodipine (n=1)	P (n=1)
			sertraline (n=1)	P/G (n=1)
			zopiclone (n=1)	P/G (n=1)
	Drug dosage adjusted	10 (22)	felodipine (n=1)	P (n=1)
			insulin (n=3)	P (n=1); P/G (n=2)
			metformin (n=2)	P (n=1); P/G/F (n=1)

For peer review only

		metoprolol (n=1)	P (n=1)
		propiomazine (n=1)	P/G (n=1)
		repaglinide (n=1)	P/G/F (n=1)
		sertraline (n=1)	P/G (n=1)
Drug added	4 (9)	omeprazole (n=1)	P/G (n=1)
		atorvastatin (n=1)	P/F (n=1)
		enalapril (n=1)	P/G/F (n=1)
		hydrochlorothiazide (n=1)	P (n=1)
		melatonin (n=1)	P/G (n=1)
		paracetamol (n=1)	P (n=1)
Other	2 (4)	enalapril (changed to other brand because of suspected side effects) (n=1)	P/G (n=1)
		metformin (compliance discussed) (n=1)	P (n=1)

- F, planned follow-up; G, treatment goal; N/A, not applicable; P, drug treatment problem

* ≥1 actions could be recorded for a single patient.

For peer review only

DISCUSSION

Main findings

In this study, we show that more than six in ten patients in primary care had appropriate drug treatment, when assessed from an overall perspective by a physician, taking morbidity, life expectancy, and concurrent drug treatment into account. Only one in ten patients had suboptimal drug treatment where an immediate change was suggested to be prioritized, mostly concerning a withdrawal of sedatives/anxiolytics. For one in four patients, the suboptimal treatment was suggested to be of lower priority, predominantly concerning the withdrawal of a PPI.

According to the procedure codes in the medical records, primary care physicians documented medication reviews in about half of all older people at least annually, with focus on those of old age with extensive comorbidities and long medication lists. For one in three

For peer review only - http://bmjopen.bmj.com/site/about/guidelines.xhtml

patients, the medication review resulted in an active change of treatment, most often a dosage adjustment as, for example, increasing the metformin dose due to high glycated hemoglobin.

Strengths and limitations

To the best of our knowledge this is the first study to provide information on the quality of drug treatment in older people in primary care from a broad family physician perspective, taking individual factors and priorities in the complex clinical situation into account. Thereby, our pilot study contributes valuable information for sample size consideration in future studies on prescribing practices and interventions for improved drug treatment. Another strength is that the physician assessment was preceded by extensive screening for PIMs and PPOs, thus minimizing the risk of overlooking important aspects regarding the prescribing quality. Indeed, only one in twenty patients did not have any PIMs/PPOs, indicating that our screening tools captured potentially suboptimal treatment to a greater extent than many other studies.[4,5,17] An additional strength is the fact that only one patient was excluded, an important aspect for generalizability.

Limitations of this study include the sample size and the fact that data were obtained from one health care centre only. This may reduce the external validity as centres may differ from each other regarding, for instance, population covered and prescribing traditions.[24] However, a limited sample size may be justified in a pilot study; a full-scale study, sized on the basis of these pilot results, has been initiated. Another limitation is that the physician assessments were performed by one person and not a team. However, this physician had relevant expertise as well as previous experience of scientifically assessing pharmacotherapy in older people.[23] In addition, ambiguities in the assessments were discussed with a senior clinical pharmacologist. Nevertheless, full-scale studies should preferably include two assessors and

For peer review only - http://bmjopen.bmj.com/site/about/guidelines.xhtml

consensus discussions to provide information on inter-rater agreement. Another limitation is that the study was restricted to information in the medical records from primary care. Indeed, the assessments were based on available data in routine care, carrying a risk that the information about the medical history, diagnoses, examinations, and laboratory tests had not been documented or appropriately coded in the medical records. Yet, this data source is more comprehensive than drug registers, either alone or linked to other registers, which are frequently used in studies on prescribing practices.[25]

Interpretation

The apparent discrepancy between the prevalence of suboptimal drug treatment according to the physician assessment and the screening tools illustrates the importance of taking future research on prescribing practices a step further to increase the medical relevance. Our results suggest that the limitations regarding the concurrent validity of general screening tools in hip fracture patients and the very old[18,26-28] may also apply to older people in general in primary care. Indeed, although the positive predictive value for several indicator sets has been reported to be acceptable when determined separately,[26,27] the PIMs/PPOs may not be relevant when taking the overall clinical picture into account, that is, from a broad family physician perspective.

The low prevalence of suboptimal drug treatment where an immediate change was suggested contributes to the understanding of the medical practice in primary care. In fact, the level of priority, according to our definition, may represent a balance between feasibility and expected patient harm; changes that can be achieved with reasonable efforts in relation to the anticipated patient benefit will get a higher priority than the opposite. Indeed, the changes suggested by the assessing physician predominantly concerned sedatives/anxiolytics and

For peer review only - http://bmjopen.bmj.com/site/about/guidelines.xhtml

PPIs, withdrawals of which may not easily be achieved once they have been initiated.[24,29] The high prevalence of PPI without an underlying indication is in agreement with a recent population-based register-based study where no underlying disease- or a drug-related reason could be identified for four in ten patients on long-term treatment with PPI.[30] Our pilot findings also contributes to the understanding of the limited implementation of recommendations for drug changes after medication reviews by third parties.[31]

Interestingly, the proportion of patients with suboptimal drug treatment was similar for patients with and without a procedure code explicitly stating that a medication review had been performed over the last year. However, patients with such a procedure code had a greater burden of disease, also illustrated by the higher number of drugs in the medication list.[32] Thus, the treatment in multimorbid patients can be expected to be more complex, and it is reassuring that drug treatment quality is acceptable to the same extent also in these patients. Although this pilot study does not allow conclusions, one may speculate that performed medication reviews may have contributed to appropriate drug treatment in multimorbid patients requiring complex treatment considerations.

Patients as defined in the Swedish regulations to receive a medication review at least annually, that is, patients ≥75 years of age with ≥5 drugs in the medication list, had this procedure code documented to a large extent. The adherence to this regulation, despite the absence of an established association between age and drug treatment quality,[18] and the limitations of cut-offs of number of drugs in the medication list to reflect quality of drug treatment,[33] may illustrate that monetary compensations can direct health care efforts to some extent. However, the evidence for effects of pay for performance policies in improving quality of care and health outcomes is limited.[34] Indeed, a recent study, investigating the

For peer review only - http://bmjopen.bmj.com/site/about/guidelines.xhtml

impact of pay for performance regarding the procedure code 'medication review' found no apparent association between this code and the prevalence of PIMs, although the number of patients with such a code increased.[35]

The actions performed within the documented medication reviews were quite few, as only one out of 14 drugs were acted upon. This finding may give the impression that medication reviews by the attending physician is a minor effort. However, as a medication review implies a medical assessment, ascertaining that the drug treatment is reasonable given the current health status, the decision not to change a drug may be just as important. Moreover, the basic part of a medication review, the medication reconciliation, was not captured in the present study.

We were happy to find that the problem underlying the treatment action was documented in the medical records in all but one case. Indeed, such information is important in transitions of care. Nevertheless, our results also suggest that documentation is an area with potential for improvement; the goal of the drug treatment change was only stated in about half of the cases, and the planned follow-up in a minority.

In conclusion, the results of this pilot study suggest that current prescribing practices in health care may be quite good, potentially due to efforts over the last decades for rational use of medicines, for example easily available information on recommended drugs,[36,37] computerized decision support,[38,39] and indicators of prescribing quality for benchmarking and educational purposes.[21,40] Indeed, although the latter may reflect quality of drug treatment to a limited extent, they may be valuable in particular for junior physicians, as they reflect expert opinions on current best practice for an average older patient. We also want to

For peer review only - http://bmjopen.bmj.com/site/about/guidelines.xhtml

emphasize that, although our findings indicate that drug treatment in older people in primary care is in general appropriate, endeavors for improvements are always desirable. Our results, reflecting drug treatment quality from a broad and clinically relevant perspective taking individual factors and medical priorities in the complex clinical situation into account, may be useful in sample size considerations for future non-randomized and randomized studies, focusing to factors associated with prescribing practices as well and interventions.

For peer review only

For peer review only - http://bmjopen.bmj.com/site/about/guidelines.xhtml

STATEMENTS

Author contributorship: N.P.L. contributed to study design, acquisition of data, analysis and interpretation of results. S.M.W. contributed to study design, analysis and interpretation of results. Both authors drafted and revised the article, and approved the final manuscript.

Conflict of Interest: The authors declare that they have no conflict of interest.

Funding: This work was supported by the Swedish Research Council; the Swedish state under the agreement between the Swedish government and the county councils, the ALF-agreement; and the Department of Research and Development in Södra Älvsborg.

Patient consent: Not required.

Ethical approval: The study was approved by the Regional Ethical Review Board in Gothenburg, Sweden (DRN: 1046-15). Informed consent was waived in the approval.

Data sharing: The datasets generated and analysed during the current study are not publicly available due to Swedish data protection laws. The data can be shared with authorized persons after approved application from the Regional Ethical Review Board in Gothenburg (https://www.epn.se/en/start/the-organisation/).

For peer review only

REFERENCES

1. Alldred DP, Kennedy MC, Hughes C, et al. Interventions to optimise prescribing for older people in care homes. *Cochrane Database Syst Rev* 2016;**2**:Cd009095.

2. Gillaizeau F, Chan E, Trinquart L, et al. Computerized advice on drug dosage to improve prescribing practice. *Cochrane Database Syst Rev* 2013(11):CD002894.

3. Patterson SM, Cadogan CA, Kerse N, et al. Interventions to improve the appropriate use of polypharmacy for older people. *Cochrane Database Syst Rev* 2014;**10**.

4. Tommelein E, Mehuys E, Petrovic M, et al. Potentially inappropriate prescribing in community-dwelling older people across Europe: a systematic literature review. *Eur J Clin Pharmacol* 2015;**71**(12):1415-27.

5. Guaraldo L, Cano FG, Damasceno GS, et al. Inappropriate medication use among the elderly: a systematic review of administrative databases. *BMC geriatrics* 2011;**11**:79.

6. Clyne W, Blenkinsopp A, Seal R. A guide to medication review. Secondary A guide to medication review [Web Page] 2008. http://www.cff.org.br/userfiles/52%20-%20CLYNE%20W%20A%20guide%20to%20medication%20review%202008.pdf.

7. Marengoni A, Onder G. Guidelines, polypharmacy, and drug-drug interactions in patients with multimorbidity. *Bmj* 2015;**350**:h1059.

8. Zhan C, Sangl J, Bierman AS, et al. Potentially inappropriate medication use in the community-dwelling elderly: findings from the 1996 Medical Expenditure Panel Survey. *Jama* 2001;**286**(22):2823.

9. The Federal Union of German Associations of Pharmacists. Grundsatzpapier zur Medikationsanalyse und zum Medikationsmanagement [Medication analysis and Medication management]. Secondary Grundsatzpapier zur Medikationsanalyse und zum Medikationsmanagement [Medication analysis and Medication management] [Web Page] 2014.

For peer review only - http://bmjopen.bmj.com/site/about/guidelines.xhtml

http://www.abda.de/fileadmin/assets/Medikationsmanagement/Grundsatzpapier_MA_MM_GBAM.pdf.

10. Holland R, Desborough J, Goodyer L, et al. Does pharmacist-led medication review help to reduce hospital admissions and deaths in older people? A systematic review and meta-analysis. *British Journal of Clinical Pharmacology* 2008;**65**(3):303-16.

11. The National Board of Health and Welfare. Senaste version av SOSFS 2000:1. Socialstyrelsens föreskrifter och allmänna råd om läkemedelshantering i hälso- och sjukvården [Medication reviews]. Secondary Senaste version av SOSFS 2000:1. Socialstyrelsens föreskrifter och allmänna råd om läkemedelshantering i hälso- och sjukvården [Medication reviews] 2016. https://lakemedelsverket.se/upload/om-lakemedelsverket/NLS/Socialstyrelsen.se.pdf.

12. Christensen M, Lundh A. Medication review in hospitalised patients to reduce morbidity and mortality. *Cochrane Database Syst Rev* 2016;**2**:Cd008986.

13. Hohl CM, Wickham ME, Sobolev B, et al. The effect of early in-hospital medication review on health outcomes: a systematic review. *Br J Clin Pharmacol* 2015;**80**(1):51-61.

14. Wallerstedt SM, Kindblom JM, Nylén K, et al. Medication reviews for nursing home residents to reduce mortality and hospitalization: systematic review and meta-analysis. *British Journal of Clinical Pharmacology* 2014;**78**(3):488.

15. Lehnbom EC, Stewart MJ, Manias E, et al. Impact of medication reconciliation and review on clinical outcomes. *The Annals of pharmacotherapy* 2014;**48**(10):1298-312.

16. Huiskes VJ, Burger DM, van den Ende CH, et al. Effectiveness of medication review: a systematic review and meta-analysis of randomized controlled trials. *BMC family practice* 2017;**18**(1):5.

For peer review only - http://bmjopen.bmj.com/site/about/guidelines.xhtml

17. Hill-Taylor B, Sketris I, Hayden J, et al. Application of the STOPP/START criteria: a systematic review of the prevalence of potentially inappropriate prescribing in older adults, and evidence of clinical, humanistic and economic impact. *J Clin Pharm Ther* 2013;**38**(5):360.

18. Lönnbro J, Wallerstedt SM. Clinical relevance of the STOPP/START criteria in hip fracture patients. *Eur J Clin Pharmacol* 2017;**73**(4):499-505.

19. O'Mahony D, O'Sullivan D, Byrne S, et al. STOPP/START criteria for potentially inappropriate prescribing in older people: version 2. *Age Ageing* 2015;**44**(2):213.

20. Renom-Guiteras A, Meyer G, Thürmann PA. The EU(7)-PIM list: a list of potentially inappropriate medications for older people consented by experts from seven European countries. *European Journal of Clinical Pharmacology* 2015;**71**(7):861.

21. Fastbom J, Johnell K. National indicators for quality of drug therapy in older persons: the Swedish experience from the first 10 years. *Drugs Aging* 2015;**32**(3):189.

22. WHO Collaborating Centre for Drug Statistics Methodology. Guidelines for ATC classification and DDD assignment 2013. Secondary WHO Collaborating Centre for Drug Statistics Methodology. Guidelines for ATC classification and DDD assignment 2013 2016/07/05/08:38:58 2012. http://www.whocc.no/filearchive/publications/1_2013guidelines.pdf.

23. Parodi López N, Villán Villán YF, Granados Menéndez MI, et al. Prescripción potencialmente inapropiada en mayores de 65 años en un centro de salud de atención primaria [Potentially inappropriate prescribing in patients over 65 years-old in a primary care health centre]. *Aten Primaria* 2014;**46**(6):290-7.

24. Hedenrud TM, Svensson SA, Wallerstedt SM. "Psychiatry is not a science like others" - a focus group study on psychotropic prescribing in primary care. *BMC Fam Pract* 2013;**14**:115.

For peer review only - http://bmjopen.bmj.com/site/about/guidelines.xhtml

25. Wallerstedt SM, Wettermark B, Hoffmann M. The First Decade with the Swedish Prescribed Drug Register - A Systematic Review of the Output in the Scientific Literature. *Basic Clin Pharmacol Toxicol* 2016;**119**(5):464-69.

26. Wallerstedt SM, Belfrage B, Koldestam A, et al. [Many patients with suboptimal drug treatment are missed. Sensitivity, specificity and prediction value of drug-specific quality indicators for the elderly]. *Lakartidningen* 2016;**113**:DSU4.

27. Wallerstedt SM, Belfrage B, Fastbom J. Association between drug-specific indicators of prescribing quality and quality of drug treatment: a validation study. *Pharmacoepidemiology and Drug Safety* 2015;**24**:906-14.

28. Dalleur O, Boland B, De Groot A, et al. Detection of potentially inappropriate prescribing in the very old: cross-sectional analysis of the data from the BELFRAIL observational cohort study. *BMC Geriatr* 2015;**15**:156.

29. Lodrup AB, Reimer C, Bytzer P. Systematic review: symptoms of rebound acid hypersecretion following proton pump inhibitor treatment. *Scand J Gastroenterol* 2013;**48**(5):515-22.

30. Wallerstedt SM, Fastbom J, Linke J, et al. Long-term use of proton pump inhibitors and prevalence of disease- and drug-related reasons for gastroprotection-a cross-sectional population-based study. *Pharmacoepidemiol Drug Saf* 2016;**16**(1):9-16.

31. Kwint HF, Bermingham L, Faber A, et al. The relationship between the extent of collaboration of general practitioners and pharmacists and the implementation of recommendations arising from medication review: a systematic review. *Drugs Aging* 2013;**30**(2):91-102.

32. Brilleman SL, Salisbury C. Comparing measures of multimorbidity to predict outcomes in primary care: a cross sectional study. *Fam Pract* 2013;**30**(2):172-8.

For peer review only

For peer review only - http://bmjopen.bmj.com/site/about/guidelines.xhtml

33. Belfrage B, Koldestam A, Sjöberg C, et al. Number of drugs in the medication list as an indicator of prescribing quality: a validation study of polypharmacy indicators in older hip fracture patients. *European Journal of Clinical Pharmacology* 2015;**71**(3):363.

34. Rashidian A, Omidvari AH, Vali Y, et al. Pharmaceutical policies: effects of financial incentives for prescribers. *Cochrane Database Syst Rev* 2015(8):Cd006731.

35. Ödesjö H, Anell A, Boman A, et al. Pay for performance associated with increased volume of medication reviews but not with less inappropriate use of medications among the elderly – an observational study. *Scand J Prim Health Care* 2017;**35**(3):271-78.

36. Bjorkhem-Bergman L, Andersen-Karlsson E, Laing R, et al. Interface management of pharmacotherapy. Joint hospital and primary care drug recommendations. *Eur J Clin Pharmacol* 2013;**69 Suppl 1**:73-8.

37. Axelsson MA, Spetz M, Mellen A, et al. Use of and attitudes towards the prescribing guidelines booklet in primary health care doctors. *BMC Clin Pharmacol* 2008;**8**:8.

38. Hellden A, Al-Aieshy F, Bastholm-Rahmner P, et al. Development of a computerised decisions support system for renal risk drugs targeting primary healthcare. *BMJ Open* 2015;**5**(7):e006775.

39. Bottiger Y, Laine K, Andersson ML, et al. SFINX-a drug-drug interaction database designed for clinical decision support systems. *Eur J Clin Pharmacol* 2009;**65**(6):627-33.

40. Rognstad S, Brekke M, Fetveit A, et al. Prescription peer academic detailing to reduce inappropriate prescribing for older patients: a cluster randomised controlled trial. *Br J Gen Pract* 2013;**63**(613):e554-62.

For peer review only - http://bmjopen.bmj.com/site/about/guidelines.xhtml

539 **Figure legends**

540 **Figure 1** Flowchart of the study population

For peer review only

For peer review only - http://bmjopen.bmj.com/site/about/guidelines.xhtml

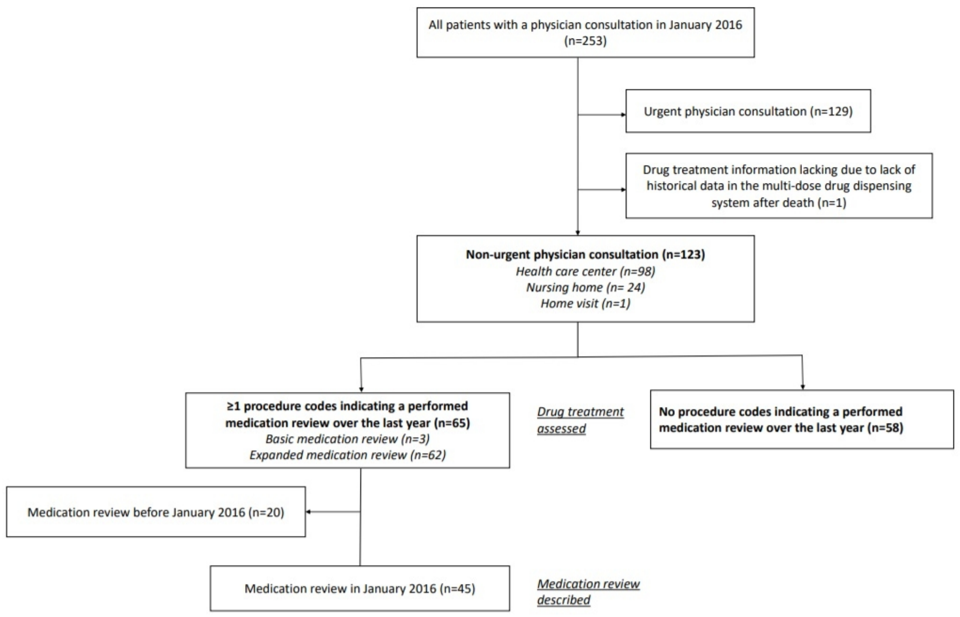

90x90mm (300 x 300 DPI)

STROBE 2007 (v4) Statement—Checklist of items that should be included in reports of *cross-sectional studies*

Section/Topic	Item #	Recommendation	Reported on page #
**Title and abstract**	1	(*a*) Indicate the study's design with a commonly used term in the title or the abstract	1,2
		(*b*) Provide in the abstract an informative and balanced summary of what was done and what was found	2,3
**Introduction**			
Background/rationale	2	Explain the scientific background and rationale for the investigation being reported	5,6
Objectives	3	State specific objectives, including any prespecified hypotheses	2,6
**Methods**			
Study design	4	Present key elements of study design early in the paper	2,6
Setting	5	Describe the setting, locations, and relevant dates, including periods of recruitment, exposure, follow-up, and data collection	2,6,7
Participants	6	(*a*) Give the eligibility criteria, and the sources and methods of selection of participants	2,7
Variables	7	Clearly define all outcomes, exposures, predictors, potential confounders, and effect modifiers. Give diagnostic criteria, if applicable	2,8,9
Data sources/ measurement	8*	For each variable of interest, give sources of data and details of methods of assessment (measurement). Describe comparability of assessment methods if there is more than one group	7,8,9
Bias	9	Describe any efforts to address potential sources of bias	15,16
Study size	10	Explain how the study size was arrived at	9,10
Quantitative variables	11	Explain how quantitative variables were handled in the analyses. If applicable, describe which groupings were chosen and why	9,10
Statistical methods	12	(*a*) Describe all statistical methods, including those used to control for confounding	9,10
		(*b*) Describe any methods used to examine subgroups and interactions	Not applicable
		(*c*) Explain how missing data were addressed	Figure 1
		(*d*) If applicable, describe analytical methods taking account of sampling strategy	Not applicable
		(*e*) Describe any sensitivity analyses	Not applicable
**Results**			

For peer review only

Participants	13*	(a) Report numbers of individuals at each stage of study—eg numbers potentially eligible, examined for eligibility, confirmed eligible, included in the study, completing follow-up, and analysed	9,10, Figure 1
		(b) Give reasons for non-participation at each stage	Figure 1
		(c) Consider use of a flow diagram	Figure 1
Descriptive data	14*	(a) Give characteristics of study participants (eg demographic, clinical, social) and information on exposures and potential confounders	10,11
		(b) Indicate number of participants with missing data for each variable of interest	Not applicable
Outcome data	15*	Report numbers of outcome events or summary measures	2, 10-14
Main results	16	(*a*) Give unadjusted estimates and, if applicable, confounder-adjusted estimates and their precision (eg, 95% confidence interval). Make clear which confounders were adjusted for and why they were included	Not applicable
		(*b*) Report category boundaries when continuous variables were categorized	10
		(*c*) If relevant, consider translating estimates of relative risk into absolute risk for a meaningful time period	Not applicable
Other analyses	17	Report other analyses done—eg analyses of subgroups and interactions, and sensitivity analyses	No applicable
**Discussion**			
Key results	18	Summarise key results with reference to study objectives	2,14
Limitations	19	Discuss limitations of the study, taking into account sources of potential bias or imprecision. Discuss both direction and magnitude of any potential bias	15,16
Interpretation	20	Give a cautious overall interpretation of results considering objectives, limitations, multiplicity of analyses, results from similar studies, and other relevant evidence	16,17,18
Generalisability	21	Discuss the generalisability (external validity) of the study results	15
**Other information**			
Funding	22	Give the source of funding and the role of the funders for the present study and, if applicable, for the original study on which the present article is based	20

*Give information separately for cases and controls in case-control studies and, if applicable, for exposed and unexposed groups in cohort and cross-sectional studies.

Note: An Explanation and Elaboration article discusses each checklist item and gives methodological background and published examples of transparent reporting. The STROBE checklist is best used in conjunction with this article (freely available on the Web sites of PLoS Medicine at http://www.plosmedicine.org/, Annals of Internal Medicine at http://www.annals.org/, and Epidemiology at http://www.epidem.com/). Information on the STROBE Initiative is available at www.strobe-statement.org.

For peer review only

BMJ Open

Quality of prescribing in older people from a broad family physician perspective: A descriptive pilot study

Journal:	*BMJ Open*
Manuscript ID	bmjopen-2018-027290.R2
Article Type:	Research
Date Submitted by the Author:	02-May-2019
Complete List of Authors:	Parodi Lopez, Naldy; Närhälsan Kungshöjd Health Centre; Sahlgrenska Academy, University of Gothenburg, Department of Pharmacology<br>Wallerstedt, Susanna; Sahlgrenska Academy, University of Gothenburg, Department of Pharmacology; Sahlgrenska universitetssjukhuset, HTA-centrum
**Primary Subject Heading**:	General practice / Family practice
Secondary Subject Heading:	Pharmacology and therapeutics
Keywords:	Aged, Inappropriate prescribing, Medication review, Pharmacoepidemiology, Prescription drugs, Primary health care

SCHOLARONE™
Manuscripts

Quality of prescribing in older people from a broad family physician perspective: A descriptive pilot study

Naldy Parodi Lopez[1,2], Susanna M. Wallerstedt[2,3]

[1]Närhälsan Vårgårda Health Centre, Sweden

[2]Department of Pharmacology, Sahlgrenska Academy, University of Gothenburg, Gothenburg, Sweden

[3]HTA-centrum, Sahlgrenska University Hospital, Gothenburg, Sweden

Corresponding author:

Naldy Parodi Lopez, Närhälsan Kungshöjd Health Centre, Kaserntorget 11A, 411 18, Gothenburg, Sweden. Tel.:+46722600348; E-mail: naldy.parodi.lopez@vgregion.se

Word count: 3939

For peer review only

For peer review only - http://bmjopen.bmj.com/site/about/guidelines.xhtml

ABSTRACT

Objectives To investigate the quality of drug treatment in older people from a broad family physician perspective, and to provide evidence for power calculations in full-scale studies on prescribing quality.

Design Descriptive, retrospective pilot study.

Setting A primary health care centre in Sweden.

Participants 123 consecutive patients, ≥65 years, with a non-urgent physician consultation in January 2016.

Measures The drug treatment was assessed by a physician as either appropriate or suboptimal, taking individual factors like morbidity, life expectancy, and concurrent drug treatment into account, and preceded by the application of 493 criteria from three screening tools for *Potentially Inappropriate Medications* (PIMs) and *Potential Prescribing Omissions* (PPOs). Suboptimal drug treatment was further categorized regarding priority: (i) immediate change suggested or (ii) actions suggested in the longer term. Prevalence of the procedure code 'medication review' and the results thereof were also recorded.

Results Median age: 76 years; 48% female. When a family physician perspective was applied, and 593 PIMs/PPOs identified in 117 (95%) patients considered, 45 (37%) patients had suboptimal drug treatment. Immediate handling was suggested in 13 (11%) patients, most often concerning withdrawals of drugs for anxiety and insomnia. Handling in the longer term was suggested in 32 (26%) patients, most often concerning overuse of proton pump inhibitors. Over the last year, the procedure code 'medication review' was recorded for 65 (53%) patients. In medication reviews recorded during January 2016 (n=45), 23 (7%) drugs out of 309 were acted upon, most often a dosage adjustment.

Conclusions This pilot study shows that when a broad family physician perspective is applied, taking individual factors and medical priorities in the complex clinical situation into

For peer review only - http://bmjopen.bmj.com/site/about/guidelines.xhtml

account, drug treatment in primary care is appropriate for the majority of older patients. The results may be useful in sample size considerations for future studies on prescribing practices.

Key words Aged; Inappropriate prescribing; Medication review; Pharmacoepidemiology; Prescription drugs; Primary health care.

For peer review only

For peer review only - http://bmjopen.bmj.com/site/about/guidelines.xhtml

STRENGTHS AND LIMITATIONS OF THIS STUDY

- This pilot study contributes current and clinically relevant information on the quality of drug treatment in older people from a broad family physician perspective, taking patient factors and medical priorities in the complex clinical situation into account, thereby providing figures for sample size consideration in full-scale studies on prescribing quality.
- To systemize the physician assessments and ensure that important potentially inappropriate medications and potential prescribing omissions were not overlooked, the assessments were preceded by the application of numerous screening tools.
- The limited sample size and the inclusion of one primary health care centre only, reduce the external validity, and full-scale studies should include two assessors to allow estimations of inter-rater agreement.

For peer review only - http://bmjopen.bmj.com/site/about/guidelines.xhtml

BACKGROUND

The purpose of drug treatment is to increase the health of patients. However, prescribing of drugs is a challenge, particularly in older people who are sensitive to drug effects and often suffer from multiple morbidities. Numerous studies have focused on describing and improving prescribing practices in this age group.[1-5]

In order to improve the quality of drug treatment, and thereby improving patient health, some countries have incorporated medication reviews in guidelines.[6-9] Frequently, medication reviews are performed by pharmacists.[3,10] In Sweden, the National Board of Health and Welfare decided in 2012 that medication reviews lie within the professional responsibilities of the physician; the only profession with a full licence to prescribe. According to national regulations, a medication review implies that the physician reconciles the drug treatment and assesses the benefit/risk for all drugs separately and combined, to ascertain that the treatment is reasonable given the current health status.[11] Medication reviews are to be documented in the medical records by a procedure code. In the Region Västra Götaland, where this study was performed, registration of this code in primary care results in monetary compensation.

Interestingly, numerous systematic reviews have failed to demonstrate effects of specifically organized medication reviews on the outcomes mortality and hospitalizations.[12-16] Provided that inappropriate prescribing is a major problem in health care,[4,5,17] this may be surprising. If, on the other hand, prescribing quality problems are less pronounced than the scientific literature may suggest, the lack of patient relevant effects may be less surprising. In this context, it is important to note that studies investigating the quality of drug treatment often focus on the prevalence of *Potentially Inappropriate Medications* (PIMs) and *Potential Prescribing Omissions* (PPOs), and three in ten PIMs as well as seven in ten PPOs have been

For peer review only

shown not to be clinically relevant at the individual level.[18] Therefore, to learn more about the quality of current prescribing practices, a physician approach, based on an overall medical assessment of the information available for a specific patient, would add important information.

Prioritizations may be an additional aspect of importance when evaluating prescribing quality concerns. As older people often have a complex clinical situation, physicians in constraints of time may have to prioritize the most urgent needs of the patient. To the best of our knowledge, this perspective has not previously been described in the scientific literature. Further, as far as we are aware, drug treatment changes upon, and documentation of, medication reviews by the attending physicians in daily care, have not previously been described.

The aim of this pilot study, with the underlying goal to provide evidence for power calculations in full-scale studies on prescribing quality, was to investigate the quality of drug treatment in older people from a broad family physician perspective, taking individual factors and medical priorities in the complex clinical situation into account. We also wanted to describe prescribing practices by physicians in primary care, with focus on medication reviews as performed according to a procedure code in the medical record.

MATERIALS AND METHODS

Design and setting

A descriptive pilot study was performed in a primary health care centre belonging to the Swedish National Health Service in Region Västra Götaland. The centre serves approximately 10,000 patients and is staffed by about 10 physicians (5 specialists in family

For peer review only - http://bmjopen.bmj.com/site/about/guidelines.xhtml

medicine, 3 residents in family medicine, 2 licensed physicians without specialist competence, and one intern). Two nursing homes, with approximately 100 residents, are attached to the centre.

Participants

All patients, ≥65 years of age, with a non-urgent consultation registered in a primary health care centre in January 1st to January 31st 2016, were included in the study. Thus, visits in the health care centre as well as visits in nursing homes and home visits were consecutively included if not caused by an urgent event. If a patient had >1 consultation during the study period, the first one, as well as the information available prior to this visit, was included in the study.

Data source

Data were retrospectively and manually extracted from electronic medical records (Asynja Visph and Journal III). The centre physicians were not informed beforehand that their work would be monitored the month in question. For each patient, socio-demographic and health-related data were retrieved including age, sex, residence, cognition, multi-dose drug dispensing (machine-dispensed unit bags with drugs that should be ingested concomitantly, for patients who have difficulties in handling their medications), drug treatment, and morbidities. Regarding morbidities, we recorded the presence of medical conditions appearing in the Screening Tool of Older Persons´ potentially inappropriate Prescriptions (STOPP), the Screening Tool to Alert to Right Treatment (START),[19] the EU(7)-PIM list,[20] and the set of indicators of prescribing quality provided by the Swedish National Board of Health and Welfare.[21]

For peer review only - http://bmjopen.bmj.com/site/about/guidelines.xhtml

For each drug in the current medication list, at the end of the visit according to the medical records including electronic prescriptions with detailed information on prescribed products and doses, we recorded the substance name, the Anatomic Therapeutic Classification code (ATC),[22] and information as to whether the drug was prescribed for regular use or as needed. Drugs for external use were included only if having potential systemic effects. Combination drugs were counted as one drug. Two drugs including the same substance and formulation, for example two different dosages of the same drug, were counted as one drug. The number of drugs that were used regularly (i.e. not for a temporary condition such as an infection) was recorded.

We recorded if a procedure code, explicitly stating that a medication review had been performed, was present or absent in the medical records of the physician consultation in January 2016. If present, we recorded the actions taken and the content of the related documentation, based on the information available in the medical records and the electronic prescriptions. Thus, each drug in the medication list was categorized as unchanged, withdrawn, dosage adjusted, added, or other. The changes in drug treatment were also summated at the patient level, categorized as ≥1 drugs being withdrawn, dosage adjusted, added, or other. Further, we recorded how the actions were documented in the medical records, focusing on whether the drug treatment problem was described, the goal with the treatment mentioned, and planned follow-up expressed. In order to elucidate to what extent the procedure code medication review is documented at least annually, we extended the search in the medical record for this action, if not recorded in January 2016, to the entire year preceding the physician consultation. The contents of these medication reviews were not further analysed, as the focus of the study was drug treatment as of January 2016.

For peer review only - http://bmjopen.bmj.com/site/about/guidelines.xhtml

Assessments

From a family physician perspective

For every patient, the quality of the drug treatment was assessed at the overall level, from the perspective of a physician specialized in family medicine (NPL), with experience of assessing drug treatment quality in older people,[23] taking into account individual characteristics of the patient such as morbidity, life expectancy, and concurrent drug treatment. The assessments were based on the information in the electronic medical records and the assessor's clinical experience. The assessor categorized each patient's drug treatment as either *appropriate* or *suboptimal*. Suboptimal treatment was further categorized according to the suggested handling of the drug treatment change: *high priority* if the suggestion was to change ≥1 drugs immediately, and *low priority* if changes could be handled in the longer term. In cases of doubt concerning the assessments, a senior specialist physician in clinical pharmacology (SMW) was consulted, both authors had a discussion, and came to a consensus decision. Information on suboptimal treatment prioritized for an immediate action was forwarded to the physician who attended the patient.

Application of screening tools prior to physician assessment

In order to systemize the physician assessments and ensure that important PIMs and PPOs were not overlooked, the overall assessment of each patient was preceded by the manual application of a total of 493 criteria from three established screening tools: the STOPP/START criteria version 2, consisting of 80 PIMs and 34 PPOs,[19] the EU(7)-PIM list consisting of 282 PIMs,[20] and the Swedish indicator set in which we used 97 out of 98 drug- and diagnosis-specific indicators of PIMs (n=77) as well as rational (n=20) treatments/treatment strategies;[21] one diagnosis-specific indicator was excluded because it was not applicable at the individual level. To comply with the other indicator sets, we

For peer review only - http://bmjopen.bmj.com/site/about/guidelines.xhtml

recorded the absence of a rational treatment/treatment strategy in the Swedish indicator set as a PPO. The sets partly overlapped as, for example, potential overuse of proton pump inhibitors and long-acting benzodiazepines were alerted in all sets. Renal function, reflected in the estimated glomerular filtration rate (eGFR), was extracted from the medical records. For patients where an eGFR was not provided in this source, we calculated creatinine clearance according to the Cockcroft-Gault equation. The assessing physician had a hard copy with all criteria, and all detected PIMs/PPOs were marked for each patient. The assessor was also allowed to identify inappropriate drug treatment not covered by the screening tools.

Statistics

SPSS (IBM SPSS Statistics for Windows, Version 19.0, Armonk, NY) was used for descriptive analyses concerning characteristics of patients and drug treatment by the absence/presence of ≥1 procedure codes for medication review over the last year. In the sample size considerations for this pilot study, we considered that including all patients fulfilling our inclusion criteria during one month, anticipated to amount to about a hundred, would yield useful results for the power calculations in the full-scale study, i.e. reasonably certain prevalence figures on suboptimal drug treatment as well as information on the prevalence of documented medication reviews. Values are presented as numbers (percentages) as well as mean ± standard deviation (SD) or median and interquartile range (IQR) or range where appropriate. As the Swedish regulations declare that physicians shall perform annual medication reviews for persons ≥75 years of age with ≥5 drugs in the medication list, these categories were also used.

Patient and public involvement

For peer review only - http://bmjopen.bmj.com/site/about/guidelines.xhtml

This research was done without patient involvement. Patients were not invited to comment on the study design and were not consulted to develop patient relevant outcomes or interpret the results. Patients were not invited to contribute to the writing or editing of this document for readability or accuracy.

RESULTS

A flowchart of the study population is presented in Figure 1. A total of 123 patients were included in the analyses, 59 (48%) of whom were female (Table 1). The median age was 76 years, ranging from 65 to 102 years. The number of chronic drugs was 5 (IQR: 3-7). Impaired cognition including dementia was found in 24 (20%) patients, and 24 (20%) individuals were nursing home residents. The most common medical conditions were hypertension (n=89; 72%), chronic pain (n=50; 41%), type 2 diabetes mellitus (n=39; 32%), and osteoarthritis (n=35; 28%).

Table 1 Characteristics of patients according to the presence/absence of ≥1 procedure codes indicating a performed medication review over the last year.

		≥1 procedure codes for a medication review over the last year	
		Yes (n=65)	No (n=58)
Age	Years	79 (73-84)	73 (68-79)
	≥75 years	45 (69)	19 (33)
Female sex		30 (46)	29 (50)
Impaired cognition, including dementia		16 (25)	8 (14)
Nursing home resident		15 (23)	9 (16)
Multi-dose drug dispensing		11 (17)	6 (10)
Common morbidities in the sample	Hypertension	54 (83)	35 (60)
	Type 2 diabetes mellitus	34 (52)	5 (9)
	Chronic pain	30 (46)	20 (35)
	Gastro-oesophageal reflux	17 (26)	10 (17)

For peer review only

For peer review only - http://bmjopen.bmj.com/site/about/guidelines.xhtml

	disease or peptic ulcer disease		
	Osteoarthritis	16 (25)	19 (33)
	Insomnia	13 (20)	13 (22)
	Cardiovascular disease	12 (19)	8 (14)
	eGFR<60 ml/min	12 (19)	6 (10)
	Dementia	11 (17)	5 (9)
	Depression	11 (17)	11 (19)
	Anxiety	9 (14)	10 (17)
Number of drugs post-visit	Total	7 (6-10)	5 (3-6)
	Regular	7 (5-8)	4 (2-6)
	As needed	1 (0-2)	1 (0-1)
	≥5 drugs	54 (83)	30 (52)

- Values are presented as median (interquartile range), or number of individuals (percentage).

eGFR, estimated Glomerular Filtration Rate.

In all, the medication lists of all patients contained 773 drugs. The most commonly prescribed drugs were paracetamol (44 patients; 34%), acetylsalicylic acid (38 patients; 30%), atorvastatin (29 patients; 24%), enalapril (29 patients; 24%), cyanocobalamin (28 patients; 23%), and metformin (27 patients; 22%).

According to the physician assessment, 78 (63%) patients had *appropriate* treatment and 45 (37%) patients had *suboptimal* drug treatment, that is, a change was suggested (Table 2). In 13 patients (11% of all; 29% of those with suboptimal treatment), the suggested change in drug treatment was of high priority, i.e. suggested to take place immediately, and concerned mainly withdrawal of propiomazine (an atypical antipsychotic agent with sedative properties used for insomnia) and hydroxyzine (a first-generation antihistamine primarily used for anxiety). In the 32 remaining cases (26% of all, 71% of those with suboptimal treatment), the suggested change was of lower priority, that is, the problem/s/ was suggested to be handled in

For peer review only

the longer term, and concerned mainly potential overtreatment with proton pump inhibitors (PPI).

Table 2 Suggested drug changes in patients with suboptimal treatment according to the physician assessment.

Priority	Suggestion	Drug (n)
High, change immediate	Withdrawal	propiomazine (n=5), hydroxyzine (n=4), diazepam (n=1), naproxen (n=1), nifedipine (n=1), orphenadrine (n=1), tramadol (n=1)
Low, reconsider in the longer term	Withdrawal, apparent indication lacking or probably inappropriate treatment	PPI (n=18), cyanocobalamin (n=2), metformin (n=2), acetylcysteine (n=1), acetylsalicylic acid (n=1), biperiden (n=1), diclofenac (n=1), dipyridamole (n=1), furosemide (n=1), laxative (n=1), potassium (n=1), quetiapine (n=1), risperidone (n=1), sotalol (n=1), zopiclone (n=1) felodipine/ carbamazepine interaction (n=1)
	Addition, absence of recommended treatment	bisphosphonate (n=1), laxatives (n=1)

- PPI, proton pump inhibitors.

The application of screening tools prior to the physician assessment resulted in the identification of 593 PIMs/PPOs (median: 4; IQR: 2-7), partly overlapping, in 117 (95%) patients. STOPP identified 125 PIMs in 59 (48%) patients (median: 2; IQR: 1-3), and START identified 54 PPOs in 49 (40%) patients (median: 1; IQR: 1-1). The EU(7)-PIM list identified PIMs in 58 (47%) patients (median: 1; IQR: 1-2). The Swedish indicator set identified 96 PIMs in 48 (39%) patients (median: 2; IQR: 1-3) and 226 PPOs in 105 (85%) patients (median: 2; IQR: 1-3).

A total of 65 patients (53%) had ≥1 procedure codes stating that a medication review had been performed over the last year. Among those ≥75 years of age with ≥5 drugs in the

For peer review only - http://bmjopen.bmj.com/site/about/guidelines.xhtml

medication list (n=53), 41 (77%) patients had ≥1 procedure codes registered regarding a medication review performed over the last year. The proportion of patients with suboptimal drug treatment according to the physician assessment was equally distributed between patients with and without a procedure code for a medication review performed over the last year: 24 (37%) and 21 (36%), respectively. Six of 13 patients with suboptimal treatment of high priority had a procedure code in the medical record explicitly stating that the drug treatment had been reviewed at least once over the last year. Among 32 patients with suboptimal treatment with low priority, 18 had ≥1 procedure codes regarding a medication review over the last year.

For 15 (33%) out of 45 patients with a procedure code for a medication review in January 2016, ≥1 actions were documented in the medical record, most often a dosage adjustment (Table 3). These actions concerned 23 (7%) drugs out of 309 present in the medication lists of these patients, and a total of 14 active substances. In all, three actions were comprehensively documented in the medical records regarding the underlying problem, the treatment goal, and the planned follow-up. There were no changes in drug treatment due to reduced renal function.

Table 3 Actions taken and contents of documentation in the medical records during documented medication reviews in January 2016 (n=45).

		Patients* n (%)	Drugs involved	Documentation in the medical records
Actions taken	Drug withdrawn	3 (7)	omeprazole (n=2)	P/G (n=1); F (n=1)
			felodipine (n=1)	P (n=1)
			sertraline (n=1)	P/G (n=1)
			zopiclone (n=1)	P/G (n=1)
	Drug dosage adjusted	10 (22)	felodipine (n=1)	P (n=1)
			insulin (n=3)	P (n=1); P/G (n=2)
			metformin (n=2)	P (n=1); P/G/F (n=1)

For peer review only - http://bmjopen.bmj.com/site/about/guidelines.xhtml

		metoprolol (n=1)	P (n=1)
		propiomazine (n=1)	P/G (n=1)
		repaglinide (n=1)	P/G/F (n=1)
		sertraline (n=1)	P/G (n=1)
Drug added	4 (9)	omeprazole (n=1)	P/G (n=1)
		atorvastatin (n=1)	P/F (n=1)
		enalapril (n=1)	P/G/F (n=1)
		hydrochlorothiazide (n=1)	P (n=1)
		melatonin (n=1)	P/G (n=1)
		paracetamol (n=1)	P (n=1)
Other	2 (4)	enalapril (changed to other brand because of suspected side effects) (n=1)	P/G (n=1)
		metformin (compliance discussed) (n=1)	P (n=1)

- F, planned follow-up; G, treatment goal; N/A, not applicable; P, drug treatment problem

* ≥1 actions could be recorded for a single patient.

For peer review only

DISCUSSION

Main findings

In this study, we show that more than six in ten patients in primary care had appropriate drug treatment, when assessed from an overall perspective by a physician, taking morbidity, life expectancy, and concurrent drug treatment into account. Only one in ten patients had suboptimal drug treatment where an immediate change was suggested to be prioritized, mostly concerning a withdrawal of sedatives/anxiolytics. For one in four patients, the suboptimal treatment was suggested to be of lower priority, predominantly concerning the withdrawal of a PPI.

According to the procedure codes in the medical records, primary care physicians documented medication reviews in about half of all older people at least annually, with focus on those of old age with extensive comorbidities and long medication lists. For one in three

For peer review only - http://bmjopen.bmj.com/site/about/guidelines.xhtml

patients, the medication review resulted in an active change of treatment, most often a dosage adjustment as, for example, increasing the metformin dose due to high glycated hemoglobin.

Strengths and limitations

To the best of our knowledge this is the first study to provide information on the quality of drug treatment in older people in primary care from a broad family physician perspective, taking individual factors and priorities in the complex clinical situation into account. Thereby, our pilot study contributes valuable information for sample size consideration in future studies on prescribing practices and interventions for improved drug treatment. Another strength is that the physician assessment was preceded by the application of numerous screening tools for PIMs and PPOs, thus minimizing the risk of overlooking important aspects regarding the prescribing quality. Indeed, only one in twenty patients did not have any PIMs/PPOs, indicating that our screening tools captured potentially suboptimal treatment to a greater extent than many other studies.[4,5,17] An additional strength is the fact that only one patient was excluded, an important aspect for generalizability.

Limitations of this study include the sample size and the fact that data were obtained from one health care centre only. This may reduce the external validity as centres may differ from each other regarding, for instance, population covered and prescribing traditions.[24] However, a limited sample size may be justified in a pilot study; a full-scale study, sized on the basis of these pilot results, has been initiated. Another limitation is that the physician assessments were performed by one person and not a team. However, this physician had relevant expertise as well as previous experience of scientifically assessing pharmacotherapy in older people.[23] In addition, ambiguities in the assessments were discussed with a senior clinical pharmacologist. Nevertheless, full-scale studies should preferably include two assessors and

For peer review only - http://bmjopen.bmj.com/site/about/guidelines.xhtml

consensus discussions to provide information on inter-rater agreement. Another limitation is that the study was restricted to information in the medical records from primary care. Indeed, the assessments were based on available data in routine care, carrying a risk that the information about the medical history, diagnoses, examinations, and laboratory tests had not been documented or appropriately coded in the medical records. Yet, this data source is more comprehensive than drug registers, either alone or linked to other registers, which are frequently used in studies on prescribing practices.[25]

Interpretation

The apparent discrepancy between the prevalence of suboptimal drug treatment according to the physician assessment and the screening tools illustrates the importance of taking future research on prescribing practices a step further to increase the medical relevance. Our results suggest that the limitations regarding the concurrent validity of general screening tools in hip fracture patients and the very old[18,26-28] may also apply to older people in general in primary care. Indeed, although the positive predictive value for several indicator sets has been reported to be acceptable when determined separately,[26,27] the PIMs/PPOs may not be relevant when taking the overall clinical picture into account, that is, from a family physician perspective.

The low prevalence of suboptimal drug treatment where an immediate change was suggested contributes to the understanding of the medical practice in primary care. In fact, the level of priority, according to our definition, may represent a balance between feasibility and expected patient harm; changes that can be achieved with reasonable efforts in relation to the anticipated patient benefit will get a higher priority than the opposite. Indeed, the changes suggested by the assessing physician predominantly concerned sedatives/anxiolytics and

For peer review only - http://bmjopen.bmj.com/site/about/guidelines.xhtml

PPIs, withdrawals of which may not easily be achieved once they have been initiated.[24,29] The high prevalence of PPI without an underlying indication is in agreement with a recent population-based register-based study where no underlying disease- or a drug-related reason could be identified for four in ten patients on long-term treatment with PPI.[30] Our pilot findings also contributes to the understanding of the limited implementation of recommendations for drug changes after medication reviews by third parties.[31]

Interestingly, the proportion of patients with suboptimal drug treatment, irrespective of priority, was similar for patients with and without a procedure code explicitly stating that a medication review had been performed over the last year. However, patients with such a procedure code had a greater burden of disease, also illustrated by the higher number of drugs in the medication list.[32] Thus, the treatment in multimorbid patients can be expected to be more complex, and it is reassuring that drug treatment quality is acceptable to the same extent also in these patients. Although this pilot study does not allow conclusions, one may speculate that performed medication reviews may have contributed to appropriate drug treatment in multimorbid patients requiring complex treatment considerations.

Patients as defined in the Swedish regulations to receive a medication review at least annually, that is, patients ≥75 years of age with ≥5 drugs in the medication list, had this procedure code documented to a large extent. The adherence to this regulation, despite the absence of an established association between age and drug treatment quality,[18] and the limitations of cut-offs of number of drugs in the medication list to reflect quality of drug treatment,[33] may illustrate that monetary compensations can direct health care efforts to some extent. However, the evidence for effects of pay for performance policies in improving quality of care and health outcomes is limited.[34] Indeed, a recent study, investigating the

For peer review only

impact of pay for performance regarding the procedure code ‘medication review’ found no apparent association between this code and the prevalence of PIMs, although the number of patients with such a code increased.[35]

The actions performed within the documented medication reviews were quite few, as only one out of 14 drugs were acted upon. This finding may give the impression that medication reviews by the attending physician is a minor effort. However, as a medication review implies a medical assessment, ascertaining that the drug treatment is reasonable given the current health status, the decision not to change a drug may be just as important. Moreover, the basic part of a medication review, the medication reconciliation, was not captured in the present study.

We were happy to find that the problem underlying the treatment action was documented in the medical records in all but one case. Indeed, such information is important in transitions of care. Nevertheless, our results also suggest that documentation is an area with potential for improvement; the goal of the drug treatment change was only stated in about half of the cases, and the planned follow-up in a minority.

In conclusion, the results of this pilot study suggest that current prescribing practices in older people in primary care is in general appropriate, especially when medical priorities are considered. Efforts for rational use of medicines over the last decades may have contributed to this finding, including easily available information on recommended drugs,[36,37] computerized decision support,[38,39] and indicators of prescribing quality for benchmarking and educational purposes.[21,40] Indeed, although the latter may reflect quality of drug treatment to a limited extent, they may be valuable in particular for junior physicians, as they reflect

For peer review only

expert opinions on current best practice for an average older patient. We also want to emphasize that, although our findings indicate that drug treatment in older people in primary care may be quite good, endeavors for improvements are always desirable. Our results, reflecting drug treatment quality from a broad and clinically relevant perspective taking individual factors and medical priorities in the complex clinical situation into account, may be useful in sample size considerations for future non-randomized and randomized studies, focusing to factors associated with prescribing practices as well and interventions.

For peer review only

For peer review only - http://bmjopen.bmj.com/site/about/guidelines.xhtml

STATEMENTS

Author contributorship: N.P.L. contributed to study design, acquisition of data, analysis and interpretation of results. S.M.W. contributed to study design, analysis and interpretation of results. Both authors drafted and revised the article, and approved the final manuscript.

Conflict of Interest: The authors declare that they have no conflict of interest.

Funding: This work was supported by the Swedish Research Council; the Swedish state under the agreement between the Swedish government and the county councils, the ALF-agreement; and the Department of Research and Development in Södra Älvsborg.

Patient consent: Not required.

Ethical approval: The study was approved by the Regional Ethical Review Board in Gothenburg, Sweden (DRN: 1046-15). Informed consent was waived in the approval.

Data sharing: The datasets generated and analysed during the current study are not publicly available due to Swedish data protection laws. The data can be shared with authorized persons after approved application from the Regional Ethical Review Board in Gothenburg (https://www.epn.se/en/start/the-organisation/).

For peer review only

REFERENCES

1. Alldred DP, Kennedy MC, Hughes C, et al. Interventions to optimise prescribing for older people in care homes. *Cochrane Database Syst Rev* 2016;**2**:Cd009095.

2. Gillaizeau F, Chan E, Trinquart L, et al. Computerized advice on drug dosage to improve prescribing practice. *Cochrane Database Syst Rev* 2013(11):CD002894.

3. Patterson SM, Cadogan CA, Kerse N, et al. Interventions to improve the appropriate use of polypharmacy for older people. *Cochrane Database Syst Rev* 2014;**10**.

4. Tommelein E, Mehuys E, Petrovic M, et al. Potentially inappropriate prescribing in community-dwelling older people across Europe: a systematic literature review. *Eur J Clin Pharmacol* 2015;**71**(12):1415-27.

5. Guaraldo L, Cano FG, Damasceno GS, et al. Inappropriate medication use among the elderly: a systematic review of administrative databases. *BMC geriatrics* 2011;**11**:79.

6. Clyne W, Blenkinsopp A, Seal R. A guide to medication review. Secondary A guide to medication review [Web Page] 2008. http://www.cff.org.br/userfiles/52%20-%20CLYNE%20W%20A%20guide%20to%20medication%20review%202008.pdf.

7. Marengoni A, Onder G. Guidelines, polypharmacy, and drug-drug interactions in patients with multimorbidity. *Bmj* 2015;**350**:h1059.

8. Zhan C, Sangl J, Bierman AS, et al. Potentially inappropriate medication use in the community-dwelling elderly: findings from the 1996 Medical Expenditure Panel Survey. *Jama* 2001;**286**(22):2823.

9. The Federal Union of German Associations of Pharmacists. Grundsatzpapier zur Medikationsanalyse und zum Medikationsmanagement [Medication analysis and Medication management]. Secondary Grundsatzpapier zur Medikationsanalyse und zum Medikationsmanagement [Medication analysis and Medication management] [Web Page] 2014.

For peer review only - http://bmjopen.bmj.com/site/about/guidelines.xhtml

http://www.abda.de/fileadmin/assets/Medikationsmanagement/Grundsatzpapier_MA_MM_GBAM.pdf.

10. Holland R, Desborough J, Goodyer L, et al. Does pharmacist-led medication review help to reduce hospital admissions and deaths in older people? A systematic review and meta-analysis. *British Journal of Clinical Pharmacology* 2008;**65**(3):303-16.

11. The National Board of Health and Welfare. Senaste version av SOSFS 2000:1. Socialstyrelsens föreskrifter och allmänna råd om läkemedelshantering i hälso- och sjukvården [Medication reviews]. Secondary Senaste version av SOSFS 2000:1. Socialstyrelsens föreskrifter och allmänna råd om läkemedelshantering i hälso- och sjukvården [Medication reviews] 2016. https://lakemedelsverket.se/upload/om-lakemedelsverket/NLS/Socialstyrelsen.se.pdf.

12. Christensen M, Lundh A. Medication review in hospitalised patients to reduce morbidity and mortality. *Cochrane Database Syst Rev* 2016;**2**:Cd008986.

13. Hohl CM, Wickham ME, Sobolev B, et al. The effect of early in-hospital medication review on health outcomes: a systematic review. *Br J Clin Pharmacol* 2015;**80**(1):51-61.

14. Wallerstedt SM, Kindblom JM, Nylén K, et al. Medication reviews for nursing home residents to reduce mortality and hospitalization: systematic review and meta-analysis. *British Journal of Clinical Pharmacology* 2014;**78**(3):488.

15. Lehnbom EC, Stewart MJ, Manias E, et al. Impact of medication reconciliation and review on clinical outcomes. *The Annals of pharmacotherapy* 2014;**48**(10):1298-312.

16. Huiskes VJ, Burger DM, van den Ende CH, et al. Effectiveness of medication review: a systematic review and meta-analysis of randomized controlled trials. *BMC family practice* 2017;**18**(1):5.

For peer review only

For peer review only - http://bmjopen.bmj.com/site/about/guidelines.xhtml

17. Hill-Taylor B, Sketris I, Hayden J, et al. Application of the STOPP/START criteria: a systematic review of the prevalence of potentially inappropriate prescribing in older adults, and evidence of clinical, humanistic and economic impact. *J Clin Pharm Ther* 2013;**38**(5):360.

18. Lönnbro J, Wallerstedt SM. Clinical relevance of the STOPP/START criteria in hip fracture patients. *Eur J Clin Pharmacol* 2017;**73**(4):499-505.

19. O'Mahony D, O'Sullivan D, Byrne S, et al. STOPP/START criteria for potentially inappropriate prescribing in older people: version 2. *Age Ageing* 2015;**44**(2):213.

20. Renom-Guiteras A, Meyer G, Thürmann PA. The EU(7)-PIM list: a list of potentially inappropriate medications for older people consented by experts from seven European countries. *European Journal of Clinical Pharmacology* 2015;**71**(7):861.

21. Fastbom J, Johnell K. National indicators for quality of drug therapy in older persons: the Swedish experience from the first 10 years. *Drugs Aging* 2015;**32**(3):189.

22. WHO Collaborating Centre for Drug Statistics Methodology. Guidelines for ATC classification and DDD assignment 2013. Secondary WHO Collaborating Centre for Drug Statistics Methodology. Guidelines for ATC classification and DDD assignment 2013 2016/07/05/08:38:58 2012. http://www.whocc.no/filearchive/publications/1_2013guidelines.pdf.

23. Parodi López N, Villán Villán YF, Granados Menéndez MI, et al. Prescripción potencialmente inapropiada en mayores de 65 años en un centro de salud de atención primaria [Potentially inappropriate prescribing in patients over 65 years-old in a primary care health centre]. *Aten Primaria* 2014;**46**(6):290-7.

24. Hedenrud TM, Svensson SA, Wallerstedt SM. "Psychiatry is not a science like others" - a focus group study on psychotropic prescribing in primary care. *BMC Fam Pract* 2013;**14**:115.

For peer review only - http://bmjopen.bmj.com/site/about/guidelines.xhtml

25. Wallerstedt SM, Wettermark B, Hoffmann M. The First Decade with the Swedish Prescribed Drug Register - A Systematic Review of the Output in the Scientific Literature. *Basic Clin Pharmacol Toxicol* 2016;**119**(5):464-69.

26. Wallerstedt SM, Belfrage B, Koldestam A, et al. [Many patients with suboptimal drug treatment are missed. Sensitivity, specificity and prediction value of drug-specific quality indicators for the elderly]. *Lakartidningen* 2016;**113**:DSU4.

27. Wallerstedt SM, Belfrage B, Fastbom J. Association between drug-specific indicators of prescribing quality and quality of drug treatment: a validation study. *Pharmacoepidemiology and Drug Safety* 2015;**24**:906-14.

28. Dalleur O, Boland B, De Groot A, et al. Detection of potentially inappropriate prescribing in the very old: cross-sectional analysis of the data from the BELFRAIL observational cohort study. *BMC Geriatr* 2015;**15**:156.

29. Lodrup AB, Reimer C, Bytzer P. Systematic review: symptoms of rebound acid hypersecretion following proton pump inhibitor treatment. *Scand J Gastroenterol* 2013;**48**(5):515-22.

30. Wallerstedt SM, Fastbom J, Linke J, et al. Long-term use of proton pump inhibitors and prevalence of disease- and drug-related reasons for gastroprotection-a cross-sectional population-based study. *Pharmacoepidemiol Drug Saf* 2016;**16**(1):9-16.

31. Kwint HF, Bermingham L, Faber A, et al. The relationship between the extent of collaboration of general practitioners and pharmacists and the implementation of recommendations arising from medication review: a systematic review. *Drugs Aging* 2013;**30**(2):91-102.

32. Brilleman SL, Salisbury C. Comparing measures of multimorbidity to predict outcomes in primary care: a cross sectional study. *Fam Pract* 2013;**30**(2):172-8.

For peer review only - http://bmjopen.bmj.com/site/about/guidelines.xhtml

33. Belfrage B, Koldestam A, Sjöberg C, et al. Number of drugs in the medication list as an indicator of prescribing quality: a validation study of polypharmacy indicators in older hip fracture patients. *European Journal of Clinical Pharmacology* 2015;**71**(3):363.

34. Rashidian A, Omidvari AH, Vali Y, et al. Pharmaceutical policies: effects of financial incentives for prescribers. *Cochrane Database Syst Rev* 2015(8):Cd006731.

35. Ödesjö H, Anell A, Boman A, et al. Pay for performance associated with increased volume of medication reviews but not with less inappropriate use of medications among the elderly – an observational study. *Scand J Prim Health Care* 2017;**35**(3):271-78.

36. Bjorkhem-Bergman L, Andersen-Karlsson E, Laing R, et al. Interface management of pharmacotherapy. Joint hospital and primary care drug recommendations. *Eur J Clin Pharmacol* 2013;**69 Suppl 1**:73-8.

37. Axelsson MA, Spetz M, Mellen A, et al. Use of and attitudes towards the prescribing guidelines booklet in primary health care doctors. *BMC Clin Pharmacol* 2008;**8**:8.

38. Hellden A, Al-Aieshy F, Bastholm-Rahmner P, et al. Development of a computerised decisions support system for renal risk drugs targeting primary healthcare. *BMJ Open* 2015;**5**(7):e006775.

39. Bottiger Y, Laine K, Andersson ML, et al. SFINX-a drug-drug interaction database designed for clinical decision support systems. *Eur J Clin Pharmacol* 2009;**65**(6):627-33.

40. Rognstad S, Brekke M, Fetveit A, et al. Prescription peer academic detailing to reduce inappropriate prescribing for older patients: a cluster randomised controlled trial. *Br J Gen Pract* 2013;**63**(613):e554-62.

For peer review only - http://bmjopen.bmj.com/site/about/guidelines.xhtml

Figure legends

Figure 1 Flowchart of the study population

For peer review only

For peer review only - http://bmjopen.bmj.com/site/about/guidelines.xhtml

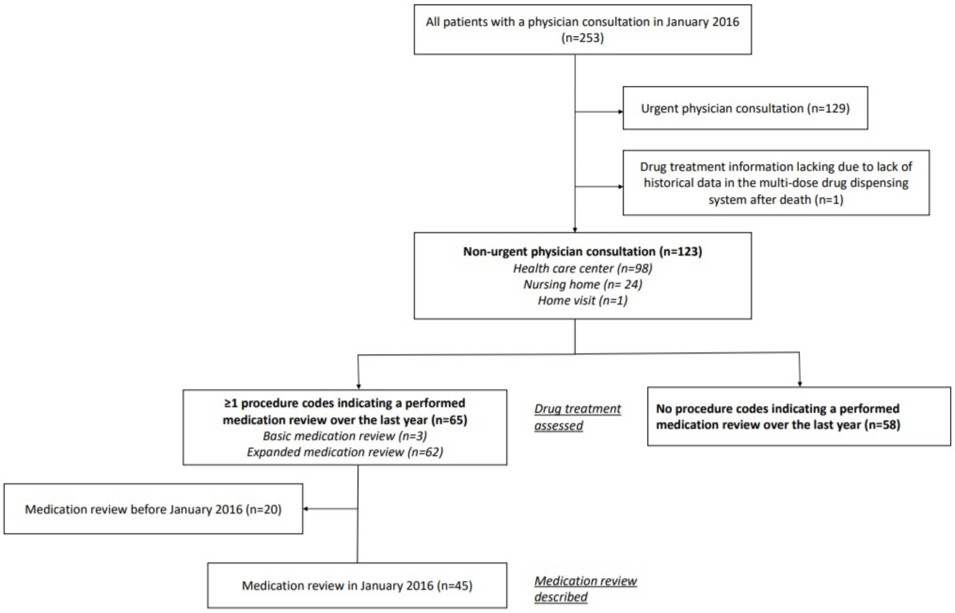

90x90mm (300 x 300 DPI)

STROBE 2007 (v4) Statement—Checklist of items that should be included in reports of *cross-sectional studies*

Section/Topic	Item #	Recommendation	Reported on page #
**Title and abstract**	1	(*a*) Indicate the study's design with a commonly used term in the title or the abstract	1,2
		(*b*) Provide in the abstract an informative and balanced summary of what was done and what was found	2,3
**Introduction**			
Background/rationale	2	Explain the scientific background and rationale for the investigation being reported	5,6
Objectives	3	State specific objectives, including any prespecified hypotheses	2,6
**Methods**			
Study design	4	Present key elements of study design early in the paper	2,6
Setting	5	Describe the setting, locations, and relevant dates, including periods of recruitment, exposure, follow-up, and data collection	2,6,7
Participants	6	(*a*) Give the eligibility criteria, and the sources and methods of selection of participants	2,7
Variables	7	Clearly define all outcomes, exposures, predictors, potential confounders, and effect modifiers. Give diagnostic criteria, if applicable	2,8,9
Data sources/ measurement	8*	For each variable of interest, give sources of data and details of methods of assessment (measurement). Describe comparability of assessment methods if there is more than one group	7,8,9
Bias	9	Describe any efforts to address potential sources of bias	15,16
Study size	10	Explain how the study size was arrived at	9,10
Quantitative variables	11	Explain how quantitative variables were handled in the analyses. If applicable, describe which groupings were chosen and why	9,10
Statistical methods	12	(*a*) Describe all statistical methods, including those used to control for confounding	9,10
		(*b*) Describe any methods used to examine subgroups and interactions	Not applicable
		(*c*) Explain how missing data were addressed	Figure 1
		(*d*) If applicable, describe analytical methods taking account of sampling strategy	Not applicable
		(*e*) Describe any sensitivity analyses	Not applicable
**Results**			

For peer review only

Participants	13*	(a) Report numbers of individuals at each stage of study—eg numbers potentially eligible, examined for eligibility, confirmed eligible, included in the study, completing follow-up, and analysed	9,10, Figure 1
		(b) Give reasons for non-participation at each stage	Figure 1
		(c) Consider use of a flow diagram	Figure 1
Descriptive data	14*	(a) Give characteristics of study participants (eg demographic, clinical, social) and information on exposures and potential confounders	10,11
		(b) Indicate number of participants with missing data for each variable of interest	Not applicable
Outcome data	15*	Report numbers of outcome events or summary measures	2, 10-14
Main results	16	(*a*) Give unadjusted estimates and, if applicable, confounder-adjusted estimates and their precision (eg, 95% confidence interval). Make clear which confounders were adjusted for and why they were included	Not applicable
		(*b*) Report category boundaries when continuous variables were categorized	10
		(*c*) If relevant, consider translating estimates of relative risk into absolute risk for a meaningful time period	Not applicable
Other analyses	17	Report other analyses done—eg analyses of subgroups and interactions, and sensitivity analyses	No applicable
**Discussion**			
Key results	18	Summarise key results with reference to study objectives	2,14
Limitations	19	Discuss limitations of the study, taking into account sources of potential bias or imprecision. Discuss both direction and magnitude of any potential bias	15,16
Interpretation	20	Give a cautious overall interpretation of results considering objectives, limitations, multiplicity of analyses, results from similar studies, and other relevant evidence	16,17,18
Generalisability	21	Discuss the generalisability (external validity) of the study results	15
**Other information**			
Funding	22	Give the source of funding and the role of the funders for the present study and, if applicable, for the original study on which the present article is based	20

*Give information separately for cases and controls in case-control studies and, if applicable, for exposed and unexposed groups in cohort and cross-sectional studies.

Note: An Explanation and Elaboration article discusses each checklist item and gives methodological background and published examples of transparent reporting. The STROBE checklist is best used in conjunction with this article (freely available on the Web sites of PLoS Medicine at http://www.plosmedicine.org/, Annals of Internal Medicine at http://www.annals.org/, and Epidemiology at http://www.epidem.com/). Information on the STROBE Initiative is available at www.strobe-statement.org.

For peer review only
